# Novel transformer-based model for NID in fog computing environment

**DOI:** 10.1038/s41598-026-35879-7

**Published:** 2026-02-18

**Authors:** Khalil M. Abdelnaby, Ahmed Y. Khedr, Aly M. Elsemary

**Affiliations:** 1 Data Science and Artificial Intelligence Department, Faculty of Information Technology, Al-Ahliyya Amman University, Amman, 19328 Jordan; 2 Systems and Computers Engineering Department, Faculty of Engineering, Al-Azhar University, Nasr City, Cairo Egypt

**Keywords:** Network intrusion detection, Machine learning, Deep learning, Transformer, NSL-KDD dataset, Engineering, Mathematics and computing

## Abstract

In fog computing, efficient and accurate network intrusion detection (NID) is critical due to the unique security challenges of distributed architectures. This research proposes a novel Transformer-based framework for NID, leveraging advanced Transformer architectures to improve feature extraction and intrusion classification. The proposed model is intended to detect different types of attack related to the attack categories including Denial-of-Service, Probe, Remote-to-Local, and User-to-Root. The proposed model utilized both the NSL-KDD and IoT-20 datasets. The results of the conducted experiments reveal that the model achieves 100% accuracy, precision, recall, and F1-score on NSL-KDD dataset while it demonstrates 99.60% accuracy in binary classification and 95.37% in multiclass classification on IoT-20 dataset. To ensure the robustness and overfitting mitigation, the model utilized cross-validation, regularization, and adversarial testing. In addition, the inclusion of the IoT-20 dataset ensures relevance to contemporary network security challenges, while attention mechanisms and explainable AI techniques enhance interpretability and practical applicability. This study highlights the transformative potential of Transformer-based models for NID in fog computing, offering a robust, scalable, and interpretable solution for securing distributed architectures.

## Introduction

**Fig. 1:**
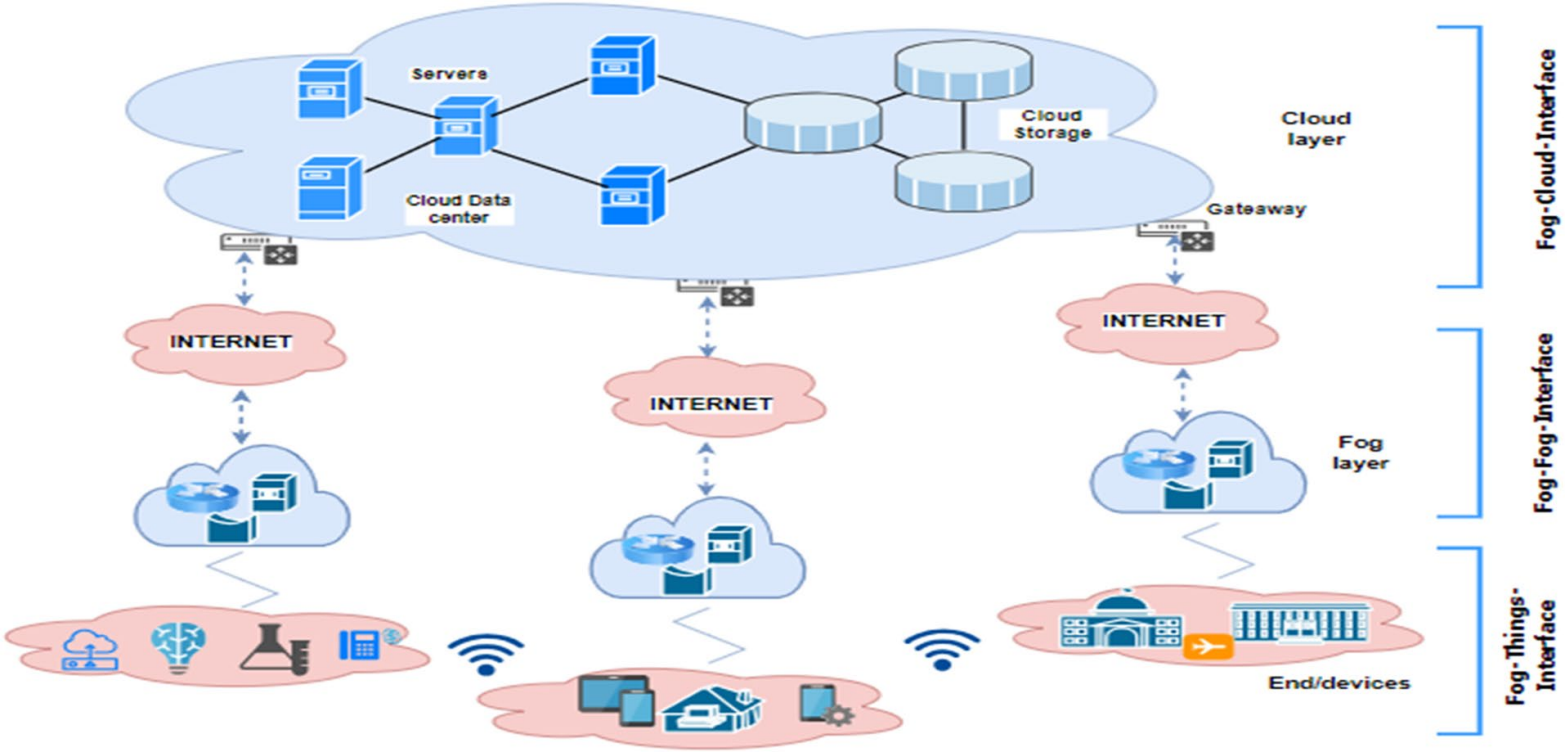
The hierarchical nature of fog computing networks.

In the era of fog computing in which data processing is decentralized and distributed across edge devices, it is essential to have robust network intrusion detection systems to recognize different types of attacks in network traffic^1^. Fog computing extends the capabilities of cloud computing by bringing computation, storage, and networking resources closer to the data source in order to enable real-time analysis and faster response in resource-constrained environments. In general, fog computing functions as a bridge between a cloud layer and an end device layer as depicted in Fig. 1. The cloud layer holds server, storage, network devices, and other necessary devices required for the cloud. On the other hand, the end/terminal device layer includes IoT devices, such as smartphones, smart vehicles, sensors, and other compact devices to monitor and sense their surroundings. The monitored and sensed data is temporary transmitted to the fog nodes or fog layer for analysis or storage purposes before reaching the cloud layer^2^.

This dynamic nature of fog computing introduces new challenges in ensuring the security and integrity of network communications^2,3^. One of the security tools to address these challenges is network intrusion detection systems (NIDS) to identify attacks in fog computing environments. Specifically, NIDs are used to continuously monitor network traffic and system logs to detect malicious activities^4^. In general, traditional rule-based intrusion detection systems face challenges in adapting to the dynamic and evolving nature of attacks. They are limited to detecting known attacks and struggle to keep up with emerging threats. In addition, the increasing volume of network traffic resulting from the Internet of Things and cloud services adds complexity to the analysis process^5^. In addition, in fog computing environment, NIDS.

must be efficient and capable of analyzing large amounts of data in real-time along with achieving fine-grained monitoring. The fine-grained monitoring is essential to attribute behavioral changes to specific network elements such as users, operating systems, and protocols. Furthermore, the diverse nature of data and protocols complicates the reliable implementation of NIDS^6^. To address these challenges, machine learning (ML) and deep learning (DL) algorithms are utilized to enhance intrusion detection in fog computing^7,8^. Even though using traditional machine learning methods has enhanced the detection of attacks in fog-based network traffic, it still has a set of shortcomings including limited labeled data, lack of interpretability, high resource demands, and difficulty adapting for evolving attacks. To overcome the underlying shortcomings, NIDs utilize the deep learning technologies.

Deep learning techniques revolutionize NIDS by automatically extracting complex patterns from network traffic and enabling detection of novel threats in fog computing environments^9,10^. Unfortunately, the powerful models deploying deep learning techniques require vast amounts of data to train and test the developed models. The required large amount of data may result in expensive computation and processing. In addition, these models lack of interpretability that could be a barrier to implement a developed NIDs in real-world scenarios. To address these issues, a Transformer model, which is a powerful advancement in machine learning, offers a promising approach to overcome challenges in machine and deep learning techniques for NIDS in the fog-computing environment^11,12^. In the context of fog computing-based NIDS, Transformer models excel in capturing intricate relationships between inputs and outputs. Unlike traditional machine learning methods, Transformer models have the ability to learn complex features autonomously from raw data, eliminating the need for manual feature engineering.

Transformer models exhibit enhanced robustness to noise and outliers in the data, a crucial factor for NIDS systems in fog computing that frequently encounter incomplete and noisy data. This enables more accurate and reliable intrusion detection in fog computing environments. In addition, Transformer models provide a solution for the shortage of labeled data required for training deep learning models in fog computing. The Transformer -based models employ either self-supervised or unsupervised learning algorithms to learn from large amount of unlabeled data that is available in fog computing environment. Accordingly, the main objective of this research paper is to investigate and analyze the utilization of a diverse range of ML and DL algorithms and introduce a novel Transformer-based model for NID in fog computing environment. In addition, the capabilities of these algorithms are utilized to improve the accuracy and efficiency of a binary/multi-class NID classifier in fog computing. Specifically, the paper encompasses several key sub-goals within the context of NID. First, it aims to conduct a comprehensive comparative evaluation of various ML algorithms including Deep Neural Network (DNN), k-Nearest Neighbor (KNN), Random Forest (RF), Extra Trees (ET), Naive Bayes (NB), Recurrent Neural Networks (RNNs), and Long-Short Term Memory (LSTM). In addition, the paper introduces a novel Transformer-based NID framework that incorporates GPT, BERT, and a full Transformer network. The developed models utilize the NSL-KDD dataset in the training and testing processes of the models. The NSL-KDD dataset contains labeled data classified into five categories: Normal, DoS, Probe, R2L, and U2R. Finally, the results of the proposed models will be compared with the state-of-the–art methods.

The paper is organized as follows: “Related work” reviews related AI research in NID focusing on the fog environment. Section “ML and DL for NID in fog computing” provides the investigation of using different ML and DL models for NID in fog computing environment. Section “Proposed transformer-based NID classification model” introduces the proposed Transformer-based model for NID in fog computing environment. Section “Experimental results” presents experimental results and “Comparison and discussion: resource efficiency for fog deployment” compares the employed AI models. Finally, “Conclusion and future work” concludes the paper and outline future work.

## Related work

This section provides an in-depth analysis of current research and advancements in the field of network intrusion detection in fog computing. Specifically, it explores traditional techniques, ML algorithms, DL techniques, and Transformer models in the domain of NID along with highlighting the strengths and limitations of different approaches.

### Traditional network intrusion detection systems

The traditional techniques used in NIDs can be categorized into misuse detection and anomaly detection. In misuse detection, the detection is performed by comparing traffic patterns against known attack signatures. On the other hand, the anomaly detection identifies traffic deviations from normal behaviour as explored in^13–19^. Aleksandar et al.^13^ compared supervised and unsupervised anomaly detection methods for network intrusion detection. They used standard and specific metrics to evaluate the employed methods on both the DARPA 1998 dataset and real-world data. Carlos et al.^14^ provided a survey on network intrusion detection methods. These methods include advanced statistical and machine learning approaches. Paul et al.^15^ introduced network intrusion detection models for misuse detection for detecting known intrusions along with anomaly detection for novel attacks. Shi et al.^16^ proposed a method to identify attack clusters in network intrusion detection systems. Their results demonstrated that their scheme is a promising solution in detecting novel attacks compared to supervised learning. Sailesh et al.^17^ provided a survey about network intrusion detection systems that covered signature-based and anomaly-detection approaches. They introduced architectures, algorithms, strengths, and limitations of each approach along with its role in cyber defence. The authors highlighted the current dominance of signature-based NIDS in addition to the potential of anomaly-detection methods in the future. Teodoro et al.^18^ explored anomaly-based intrusion detection methods, commonly used techniques, platforms, and their deployment challenges. They emphasized on the potential of the methods that detect novel attacks even though they acknowledge higher false positives compared to signature-based methods. The authors in^19^ comprehensively assessed current intrusion detection systems in terms of detection methods, approaches, technologies, and available tools such as Snort and ClamAV. In addition, they presented learned lessons, future challenges, and the importance of IDSs in cybersecurity. Even though intrusion detection scientists have being provided several traditional methods and techniques to identify attacks, these methods struggle to detect novel attacks due to the continuous trend of cyber threats getting more sophisticated. This results in emerging ML techniques into network intrusion detection in fog computing environments.

### Machine learning-based NID in fog computing

ML techniques for NIDS employ ML algorithms to learn the normal behaviour of network traffic and then identify anomalies based on measuring the traffic deviation from the learned normal behaviour. These systems are typically trained on a large dataset of historical network traffic data. Once a system is trained, it can be used to detect anomalies in real time. Cristiano Antonio et al.^20^ presented a two-step ensemble approach using reputable datasets for intrusion detection and identification in IoT and fog computing environments. The first step used an Extra Tree binary classifier for traffic analysis. If the network traffic is benign, the traffic is released. Otherwise, the network traffic proceeds into the second step to identify the specific type of attack. The second step employed an ensemble of Extra Tree, Random Forest, and DNN to identify the specific type of intrusion. The authors in^21^ introduced a hybrid approach for intrusion detection in fog-based IoT environments. The approach used a two-step process with a hybrid binary classification method called DNN-KNN. The method combines both DNN and K-nearest neighbour algorithms to achieve high accuracy and recall rates. The approach was evaluated the using the NSL-KDD and CICIDS2017 datasets. John et al.^5^ introduced an anomaly detection model in a fog-computing environment to address network security challenges. The detection model employs both genetic algorithm wrapper-based feature selection and Naive Bayes for detecting attacks. The developed model, called GANBADM, is trained and tested with NSL-KDD dataset. The model achieved high accuracy and low false positive rate in detecting network anomalies. Omar et al.^22^ presented an Effective Seeker Optimization with Machine Learning-Enabled Intrusion Detection System (ESOML-IDS) model for fog and edge computing environments. The model utilized a novel feature selection approach based on Effective Seeker Optimization (ESO) to choose an optimal set of features. In addition, it employs comprehensive learning particle swarm optimization (CLPSO) in conjunction with a Denoising Autoencoder for intrusion detection and classification. Saipriya et al.^23^ explored fog computing model as an intermediate layer between cloud and IoT devices for securing IoT sensor nodes. The paper deployed machine learning techniques in the fog layer to detect attacks on these nodes. The model used the KDDCup99 dataset for training and evaluation processes. Even though employing ML techniques in NID for fog computing environment have enhanced the attack detection compared by the traditional approaches, ML techniques face several challenges. These challenges include limited labelled data, lack of interpretability, high resource demands, and difficulty adapting to evolving attacks. In addition, adversarial attacks pose new vulnerabilities. Due to these challenges, deep learning techniques are increasingly implemented in NID for fog computing environments.

### Deep learning-based NID approaches

Deep learning techniques for NID in fog computing can automatically extract complex patterns from raw traffic data to detect not only known attacks but also novel threats. This has spurred numerous recent studies exploring deep learning techniques for NIDS including^24–37^. Abebe et al.^24^ developed a distributed attack detection scheme using a deep learning approach for the Internet of Things (IoT) ecosystem. The proposed scheme demonstrated that it is more scalable than a centralized cloud-based approach for IoT applications. Bhuvaneswari et al.^25^ proposed an anomaly detection framework for IoT traffic using a vector convolutional deep learning (VCDL) approach in a fog computing environment. The framework addressed the scalability issue of handling large-scale IoT data by distributing the training of the deep learning model across fog nodes. Muder et al.^26^ provided a deep recurrent neural network-based intrusion detection model in the fog-computing layer to detect cyber-attacks. The model used the NSL-KDD dataset for both training and evaluation of the model. Mohamed et al.^27^ introduced a deep learning intrusion detection system called Deep-IFS for Industrial IoT (IIoT) traffic. The Deep-IFS leverages LocalGRU and multi-head attention to analyze IIoT traffic sequences. The paper addressed the scalability issue by deploying Deep-IFS in a fog-computing environment and distributing training and data across worker nodes. Prabavathy et al.^28^ proposed a novel intrusion detection technique based on fog computing and Online Sequential Extreme Learning Machine (OS-ELM) to efficiently detect attacks in the highly scalable and dynamic Internet of Things (IoT) environment. The distributed architecture of fog computing enabled a distributed intrusion detection mechanism with scalability, flexibility, and interoperability. Quamar et al.^29^ used deep learning with self-taught learning for network intrusion detection. They evaluated their approach with the NSL-KDD dataset. Specifically, they compared accuracy, precision, recall, and F-measure with other previous works. Chuanlong et al.^30^ provided a deep learning intrusion detection system called RNN-IDS using RNNs. They evaluated its performance in binary and multiclass classification compared to machine learning techniques including J48, neural network, random Forest, and SVM. Wei et al.^31^ introduced HAST-IDS, a deep learning system for intrusion detection. Their proposed model automatically learns features from network traffic data and eliminates manual feature engineering. In the model, convolutional neural networks capture low-level spatial features while long-short term memory networks handle high-level temporal aspects. Chongzhen et al.^32^ proposed a deep learning approach for intrusion detection using an autoencoder-based feature extraction technique. The approach used the NSL-KDD dataset during training and testing processes. The approach aimed to identify key features from network traffic data without a decoder to potentially speed up the model training process. Ren et al.^33^ introduced a deep learning approach for intrusion detection that analyzes individual packets (not entire flows). They used word embedding to understand packet headers and long-short term memory networks to capture how features within packets relate over time for classification. Ming-Tsung et al.^34^ proposed a two-stage deep learning approach for network anomaly detection. It combines Gate Recurrent Unit (GRU) and the Denoising Auto-Encoder (DAE) models to improve accuracy. The GRU classifies flows and the flows produced with low confidence are further analyzed by the DAE for anomaly detection. Ren-hung et al.^35^ proposed an unsupervised deep learning model called D-PACK for early anomaly detection in network traffic. The D-PACK utilized a CNN and autoencoder to learn normal patterns and identify anomalies. The model focused only on initial packets of each flow for faster detection of malicious traffic. Bo et al.^36^ tackled multi-class intrusion detection and imbalanced data with a CNN-GRU model. The model incorporated hybrid sampling, feature selection, and attention fusion for improved performance. The model achieved high accuracy on benchmark datasets compared to prior CNN-GRU approaches. Salwa et al.^37^ compared various deep learning algorithms for network intrusion detection. They fine-tuned DNNs, RNNs including long-short term memory (LSTM) and Gated Recurrent Unit (GRU), CNNs, and Hybrid CNN-LSTM models using Keras Tuner. Therefore, deep learning revolutionized NIDS by automatically extracting complex patterns from network traffic and enabling detection of novel threats. Unfortunately, these powerful models require vast amounts of data that can be computationally expensive and lack of interpretability. These shortcomings make them hard to be deployed in real-world scenarios. Accordingly, the Transformer-based NID emerged into this field.

### Transformer- and attention-based NID models

Transformer models represent a specific deep learning architecture and show promise in addressing the aforementioned shortcomings resulting from applying DL techniques to NID in fog environment. They might require less data for training, offer some insights into their decision-making process and potentially be more robust to manipulation. However, deploying transformers into NIDs is still under development. A few recent studies have investigated Transformer techniques into the field of NID in fog computing environments.

Ban et al.^38^ introduced an IDS model that uses attention-based LSTMs (AT-LSTM & AT-BiLSTM) to exploit network traffic sequences for intrusion detection. They leverage the UNSW-NB15 dataset and SMOTE to handle class imbalance. Fatima Ezzahra et al.^39^ proposed an IDS model that implemented LSTMs with attention. The authors evaluated feature reduction techniques on the NSL-KDD dataset. Attention with all features and PCA with 30 components achieved the best accuracy. It reached 99.09% for binary classification while 98.49% for multiclass classification. Kanishk et al.^40^ studied whether fine-tuned Transformer models (BERT, HateBERT) can explain cyber bullying detection or not. Their models focused on text features but lack full transparency in decision-making. This highlights the need for clearer AI in content moderation. Gang et al.^41^ introduced a model combined both Transformer-Encoder and Bidirectional Long-Short Term Memory (BiLSTM) for network intrusion detection. Their approach utilized deep learning for feature extraction and achieved high performance on the NSL-KDD dataset. Hanafi et al.^42^ proposed IDSX-Attention model that combines outlier removal, dimensionality reduction, and LSTM-Attention for attack classification. It achieved significant improvements over baselines on the NSL-KDD dataset. Since the deployment of Transformer for NID in fog computing paradigm is still need to be investigated, this paper presents a comparative study of both ML and DL along Transformer models for NIDS within fog computing environments. In addition, it proposes a novel Transformer-based framework that leverages BERT, GPT, and full Transformer architectures. The framework is evaluated with the NSL-KDD dataset.

### Summary and research gap

Although intrusion detection systems based on traditional techniques, machine learning, and deep learning have achieved notable advances, important challenges remain especially when such systems are considered for deployment in fog computing environments. Signature-based and rule-driven methods are inherently limited in their ability to recognize novel or evolving attacks, while conventional machine learning approaches rely heavily on manually engineered features and often struggle to adapt to changing traffic patterns. Deep learning models alleviate some of these issues by learning richer representations, but they typically demand large volumes of labeled data, offer limited transparency in decision making, and introduce considerable computational overhead, all of which hinder their practicality at the fog layer.

More recently, attention-based and Transformer-inspired models have shown encouraging results in intrusion detection tasks. However, much of the existing research has been oriented toward sequential or text-like data representations, with relatively limited exploration of Transformers for structured, tabular network traffic in fog computing settings. In addition, prior studies often place less emphasis on deployment-critical aspects such as robustness, interpretability, resistance to adversarial manipulation, and inference efficiency factors that directly influence the feasibility of low-latency operation on resource-constrained fog nodes.

Motivated by these observations, this paper introduces a Transformer-based intrusion detection framework designed specifically for fog computing scenarios. The proposed system explores encoder-only, decoder-only, and full Transformer architectures to model global dependencies among network traffic features, while integrating explainability components and robustness analysis to enhance practical relevance. Through this design, the proposed approach offers a scalable and well-rounded alternative that meaningfully extends current state-of-the-art intrusion detection solutions.

## ML and DL for NID in fog computing

This section investigates the use of different ML and DL algorithms for NID in fog computing environment. The employed algorithms use the NSL-KDD dataset for training, evaluating, and testing different models. The dataset has several types of attack traffics along with normal traffics. For simplicity, the attack traffics are classified into four categories based on their intention functions as DoS, Probe, R2L, and U2R. Accordingly, each employed model works as binary (normal or abnormal) classifier or multiclass (normal, DoS, Probe, R2L, and U2R) classifier. In addition, each model deploys different AI techniques to measure a set of NID metrics including precision, recall, F1-Score, and accuracy. The investigated AI architectures fog computing NID employs ML and DL technologies as presented in  “Machine learning NID classification model” and “Deep learning NID classification model”; respectively.

**Fig. 2:**
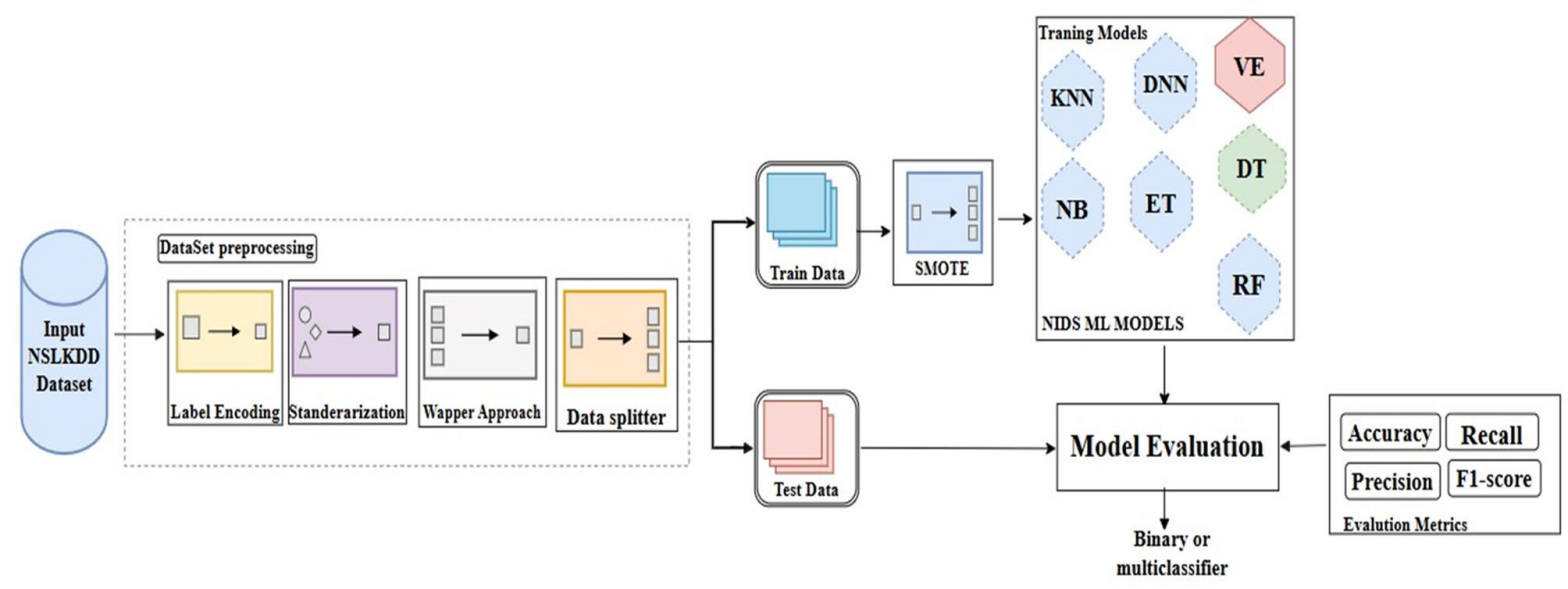
Machine learning model for NID in fog computing environment.

### Machine learning NID classification model

This section presents a machine learning model as binary and multiclass classifiers for network intrusion detection (NID) in fog computing environment. The architecture or model has three main components: dataset pre-processing component, training component, and evaluation component as depicted in Fig. 2.

**Fig. 3:**
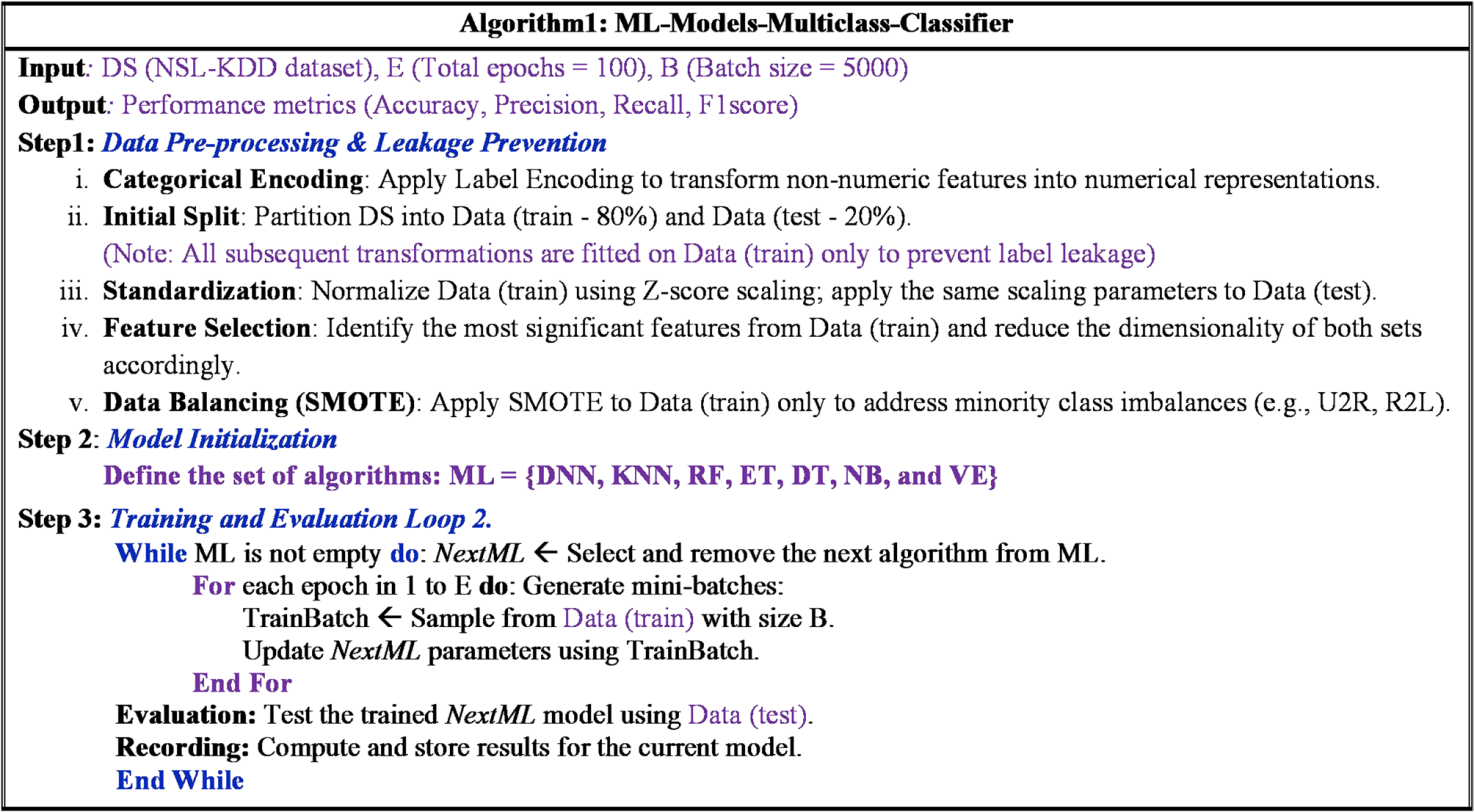
Machine learning algorithm for NID in fog computing.

The dataset pre-processing component is responsible for preparing the network traffic data in the NSL-KDD dataset to work with the training component. The dataset pre-processing component has five consecutive processes: Label encoder, Standardization, Feature selection, Data balance, and Data splitter. The Label encoder transfers the categorical features in the dataset labeled with ‘Protocol_type’, ‘Service’, and ‘Flag’ into a corresponding numerical representation to be effectively utilized in the next process^37^. 

The standardization process is responsible for removing invalid records and then standardizing the data. Standardization process helps to improve the performance of machine learning based classifiers that rely on normally distributed resources. Specifically, the standard scaling is used to normalize data based on the mean and standard deviation for each attribute in the dataset. After normalizing the data, the feature selection process selects the most relevant attribute of feature.

The process utilizes the Recursive Feature Elimination (RFE) method from the wrapper family. The RFE recursively considers smaller sets of features and makes the deployed classifier ranks the importance of each attribute. Once the feature selection process selects the most relevant features, the Data balanced process utilizes the Synthetic Minority Oversampling Technique (SMOTE) to address the disparity among different attack types. This process ensures that the models have sufficient data to understand the patterns of attacks with fewer occurrences.

**Fig. 4:**
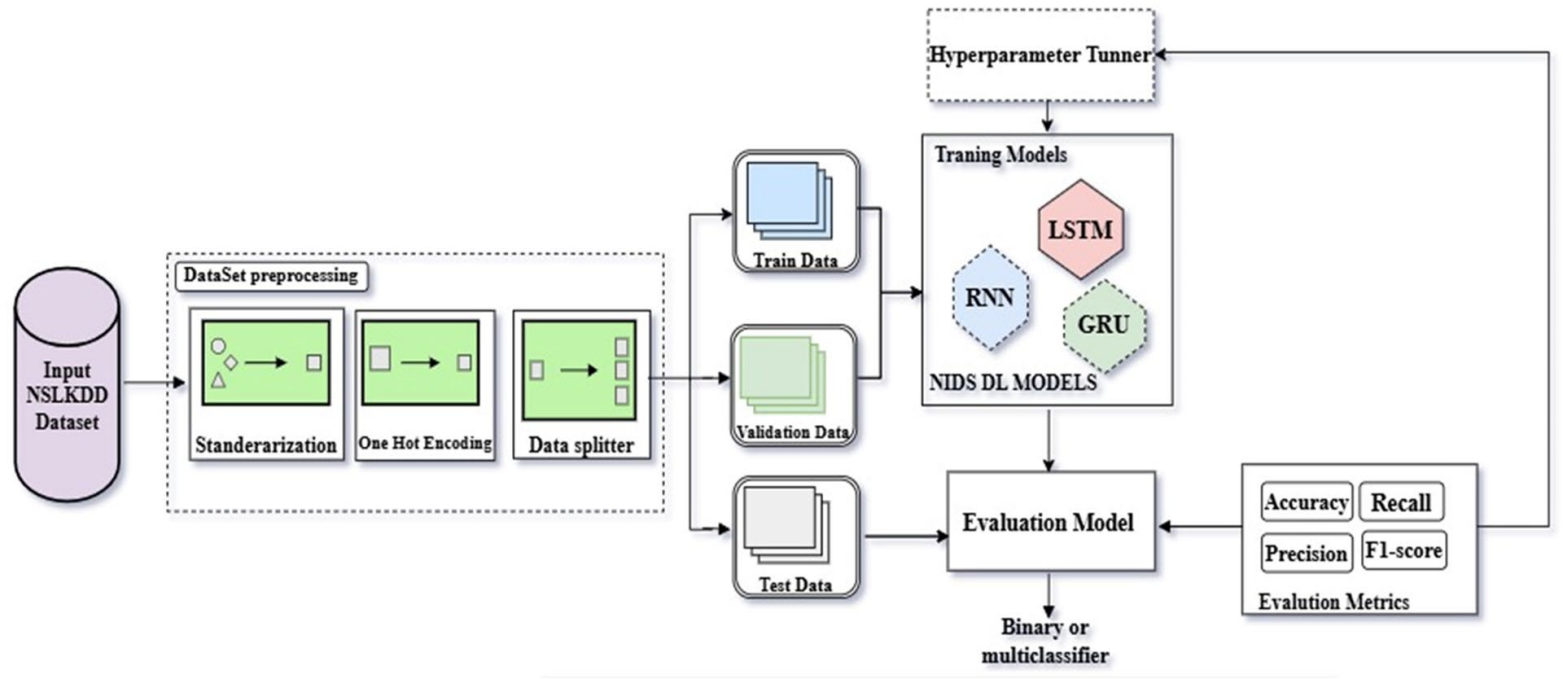
The proposed deep learning model for NID in Fog computing environment.

The SMOTE generates new instances for minority classes by creating synthetic samples based on existing examples. After balancing the data, the Data splitter process randomly divided the processed data into 80% as training data and 20% as testing data. In addition, 20% of the training data is randomly selected during the training process to evaluate the model.

The training component utilizes seven machine learning algorithms one at a time. These algorithms are Deep discriminative network (DDN), KNN, RF, ET, decision tree (DT), NB, and voting ensemble (VE). Each ML algorithm is trained with the preprocessed training data. In addition, it is evaluated with an unseen of 20% of the data training. During the training process, each ML algorithm is trained one time as a binary classifier and the other time as multiclass classifier with the associated preprocessed data^43,44^. On the other hand, the evaluation component employs precision, recall, F1-score, and accuracy as an evaluation metrics to evaluate the model during the training process and testing process. It evaluate the model with 20% of the training data during the training process to measure the learning behavior of the model while it tests the model with the testing data to measure the model performance in terms of precision, recall, F1-score, and accuracy.

Finally, the general procedure of the proposed ML model as multiclass classifier for NID in Fog computing environment is depicted in Fig. 3. The model takes the NSL-KDD dataset as an input and produces the model performance in terms of precision, recall, F1-score, and accuracy as an output. **In Step 1**, the model preprocesses the dataset DS by performing the five processes starting with the Label encoder on the DS and ending with Data splitter processes. The Data splitter divides the pre-processed data into 80% for training and 20% for testing data. The training data will be stored in the training-data variable while the testing data is kept in the testing-data variable. In addition, 20% of the training data is randomly selected for validating the model and it is stored in the variable validating-data. **In Step 2**, the employed machine learning algorithms is assigned to the list variable ML. **In Step 3**, the model repeats the process over each utilized machine learning algorithm for epoch times based on the *batchSize* value. For each algorithm, a set of operations is performed. The first operation removes the next machine learning algorithm from the set ML and assigns it to the variable *NextML*. Next, the training process is performed on the machine learning algorithm in the variable *NextML* with the *trainData* extracted from data *training-data*. Next, the evaluation process is performed on the testing data *testing-data* and then the evaluation results are recorded. This process is repeated until all machine learning algorithms are performed^45,46^.

### Deep learning NID classification model

This section details the architecture of our deep learning framework, designed to function as both a binary and multiclass classifier within the high-throughput, latency-sensitive environment of fog computing. The system is structured into three integrated stages: Data Intelligence (Pre-processing), Sequential Pattern Discovery (Training), and Performance Verification (Evaluation), as illustrated in Fig. 4.

To ensure the integrity of the experimental results and strictly prevent data leakage, the pre-processing stage follows a rigorous sequence where data partitioning occurs prior to feature transformation.

- **Data Partitioning (Data Splitter)**: Unlike traditional approaches that scale data before splitting, our framework first divides the raw dataset (DS) into training, validation, and testing sets. This ensures that the testing set remains “unseen” and that no statistical information from the test set (such as mean or variance) influences the training process.
- **Standardization**: Continuous features are scaled to a uniform range. The parameters for scaling are derived solely from the training partition and subsequently applied to the validation and test sets.
- **Categorical Mapping (One-Hot Encoding)**: Categorical attributes, including ‘Protocol_type’, ‘Service’, and ‘Flag’, are translated into unique binary vectors. For example, the ‘Protocol_type’ attribute (comprising ‘tcp’, ‘udp’, and ‘icmp’) is mapped to [1,0,0], [0,1,0], and [0,0,1], respectively.

This prevents the model from assuming a false mathematical order between categories.

The training stage evaluates three specialized architectures: Recurrent Neural Networks (RNN), Long Short-Term Memory (LSTM), and Gated Recurrent Units (GRU). These models were selected for their inherent ability to capture temporal dependencies within network traffic flows.

During training, each algorithm processes the data in batches to learn complex attack signatures. To address the severe class imbalance in datasets like NSL-KDD (e.g., the scarcity of U2R attacks), we utilized Cost-Sensitive Learning. Instead of over-sampling, we applied a weighted Cross-Entropy loss function, forcing the model to pay a higher penalty for misclassifying minority classes. This approach ensures the “learning brain” of the model remains robust across all five traffic categories.

The evaluation stage acts as the final quality gate. Each classifier is assessed against four pillars: Accuracy, Precision, Recall, and F1-Score. For the multiclass setting, the output layer utilizes a Softmax activation function to provide a probability distribution across the five classes, while the binary task utilizes a Sigmoid activation. In the context of fog computing, we specifically evaluate the trade-off between detection depth and inference latency to ensure real-time feasibility at the network edge^47–49^.

**Fig. 5:**
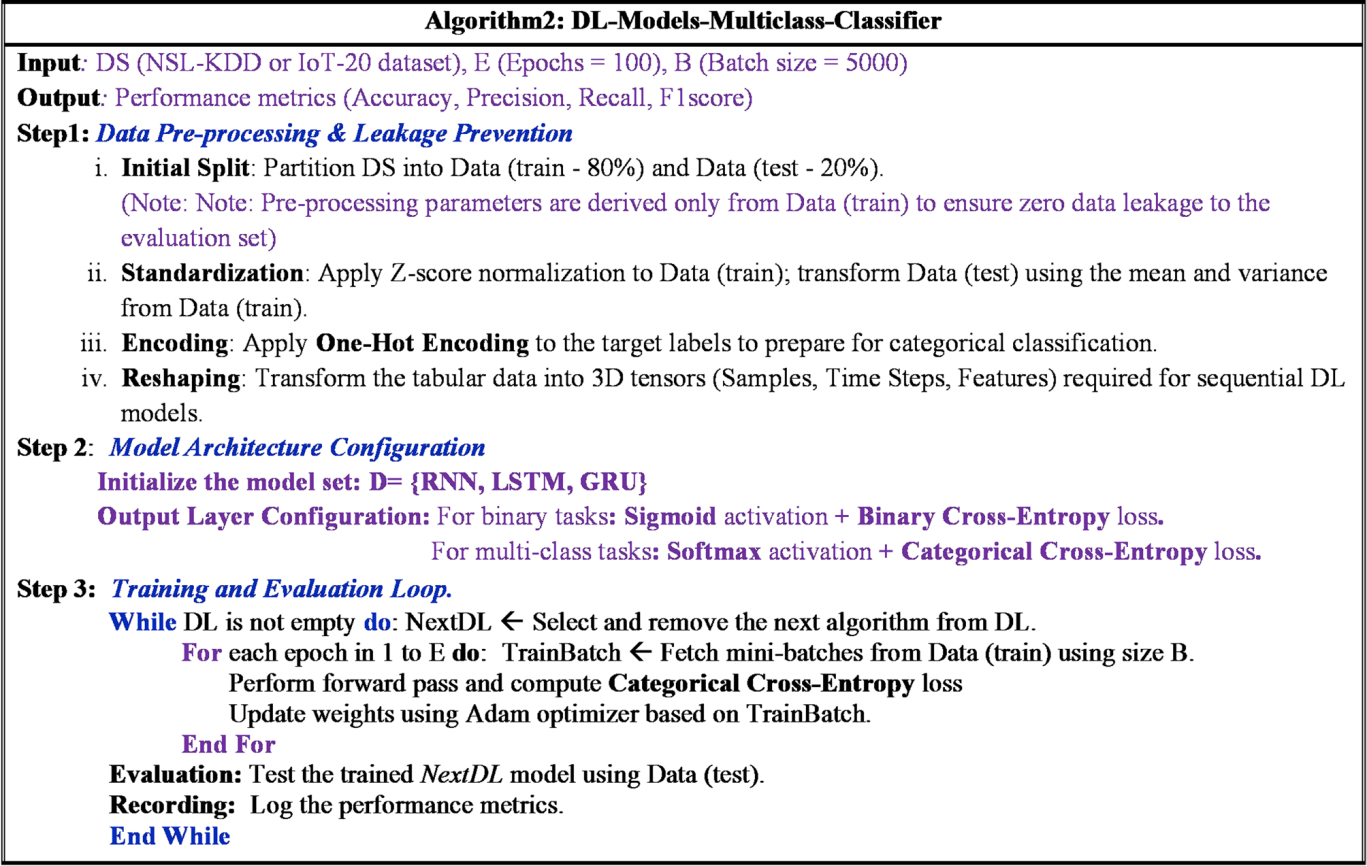
Deep learning algorithm for NID in fog computing.

The general procedure for the proposed DL multiclass classifier is formalized in Fig. 5. The operational logic follows a structured sequence: **Step 1** (Pre-processing): The system ingests the dataset (DS), performs the Data Splitter process first, followed by Standardization and One-Hot encoding. The resulting partitions are stored as training-data, validating-data, and testing-data. **Step 2** (Initialization): A list of candidate DL architectures (DL = [RNN, LSTM, GRU]) is initialized. **Step 3** (Iterative Training): The model iterates through each algorithm. For each architecture: It executes the training process using the specified BatchSize and epochs. The validating-data is monitored by a hyperparameter tuner to optimize the learning rate and prevent overfitting. **Step 4** (Final Assessment): Once training is finalized, the testing-data is introduced. The performance metrics are recorded, and the process repeats for the next architecture in the list until all evaluations are complete.

### Data pre-processing and feature encoding

To guarantee data quality and consistency, the NSL-KDD dataset was subjected to a standard pre-processing pipeline before model training. Since the NSL-KDD dataset is mostly complete but may contain placeholder or inconsistent entries, records with missing, undefined, or invalid values were first eliminated. Z-score standardization was then used to normalize continuous numerical features. To avoid data leakage, scaling parameters (mean and standard deviation) were only calculated from the training set and then applied to the validation and testing sets.

Depending on the learning paradigm, categorical features such as Protocol_type, Service, and Flag were handled differently. To avoid imposing ordinal relationships between categories, one-hot encoding was used to transform categorical attributes for both traditional machine learning and recurrent deep learning models (DNN, RNN, LSTM, and GRU). On the other hand, transformer-based models were able to capture semantic relationships between categorical values while retaining computational efficiency by mapping categorical features to low-dimensional learnable embeddings.

## Proposed transformer-based NID classification model

### Model overview

The proposed architecture is designed to adapt the strengths of sequence-modeling transformers to the domain of tabular network traffic. Unlike traditional models that treat features as a flat vector, our model captures the inter-dependencies between different network attributes by treating them as structured sequences. The model is defined by a hidden dimensionality $\:{d}_{\mathrm{m}\mathrm{o}\mathrm{d}\mathrm{e}\mathrm{l}}$ of 128 in order to guarantee high-dimensional feature representation while preserving computational efficiency for fog nodes. Each of the twelve encoder layers in the architecture makes use of four multi-head attention mechanisms. Our model uses Learned Positional Encodings instead of the fixed sinusoidal encodings used by standard natural language processing (NLP) transformers. Given the fixed order of tabular datasets like NSL-KDD and IoT-20^50,51^, this enables the Transformer to learn the precise structural importance and the relative positioning of network features during the training phase. The tabular data is converted into a format that Transformer blocks can use by the input processing layer. Each of the 41 network features in the NSL-KDD dataset is handled as a separate token inside a 41-length sequence.

- **Numerical Characteristics**: are projected using a linear embedding layer into the 128-dimensional space $\:{d}_{\mathrm{m}\mathrm{o}\mathrm{d}\mathrm{e}\mathrm{l}}$.
- **Features of Categories**: are concatenated into the sequence after first being mapped using an embedding look-up table. Regardless of their distance in the feature vector, the self-attention mechanism can evaluate the relative importance of particular network traffic flow features (such as source bytes, duration, and flag) by treating the 41 features as a series of tokens.

The proposed encoder-decoder Transformer architecture can be applied to NID in Fog environment by employing the NSL-KDD dataset as follows:

- *Input Embedding*: The input data is initially embedded into a format suitable for effectively processing the model in both the encoder and decoder.
- *Positional Embedding*: Both the encoder and decoder employ positional embedding to provide the model with information about the relative positions of tokens within the input sequence. This helps the model to capture the sequential order and temporal dependencies in the network traffic flow features data to understand the context and patterns associated with network intrusions.
- *Stacked Blocks in the Encoder*: The encoder includes stacked blocks designed to capture relevant features from the input data. Each block typically consists of (i) a self-attention mechanism that allows the model to focus on different parts of the input sequence and capture dependencies and relationships between tokens, (ii) a feedforward network that captures complex patterns in the input data, and (iii) a normalization layer to ensure proper normalization of the output.
- *Stacked Blocks in the Decoder*: The decoder also includes stacked blocks, which increase the model depth and capture complex relationships. Each block comprises (i) a masked multi-head attention mechanism that enables the model to attend the different parts of the input sequence while preventing it from attending to future positions, (ii) a feedforward network that captures further patterns in the decoder input, and (iii) a normalization layer that normalizes the output.
- *Encoder and Decoder Connection*: The encoder captures contextual information from the input data and flow this information to the encoder that generates an output based on that information.
- *Output Generation*: The output of the decoder is passed through an additional linear layer to obtain the final output. The output layer is used as binary classifier or multiclass classifier. In the binary classifier, the output layer produces either normal or abnormal while in multiclass classifier, the output layer produces one of (normal, DoS, Probe, R2L, or U2R) based on the received network traffic.

**Fig. 6:**
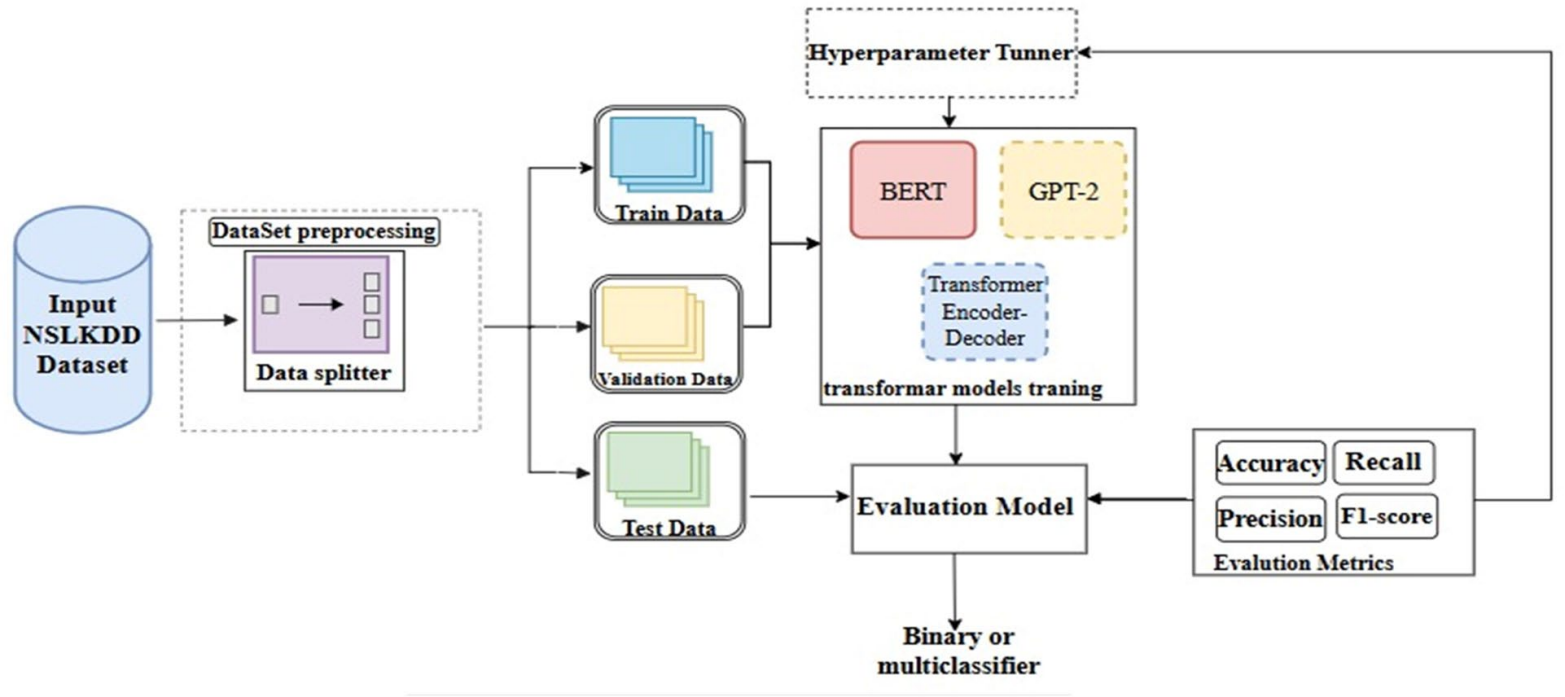
The proposed transformer-based model for NID in fog computing environment.

Accordingly, the proposed Transformer-based NID architecture in Fog computing environment comprises three main components: Data Pre-processing, Transformer training, and Evaluation, as depicted in Fig. 6. *Data Pre-processing* is responsible only for dividing the data set into training data, validating data, and testing data, as described in “Deep learning NID classification model”. The Transformer training component includes three Transformer algorithms: GPT-based NID (decoder-only), BERT-based NID (encoder-only), and full Transformer (encoder-decoder). These algorithms are adept at capturing sequential patterns. The model uses these algorithms one at a time. Each algorithm is trained on the training data subset to learn patterns and classify network traffic. Then, the performance of the model is measured with testing data in terms of precision, recall, F1-score, and accuracy.

**Fig. 7:**
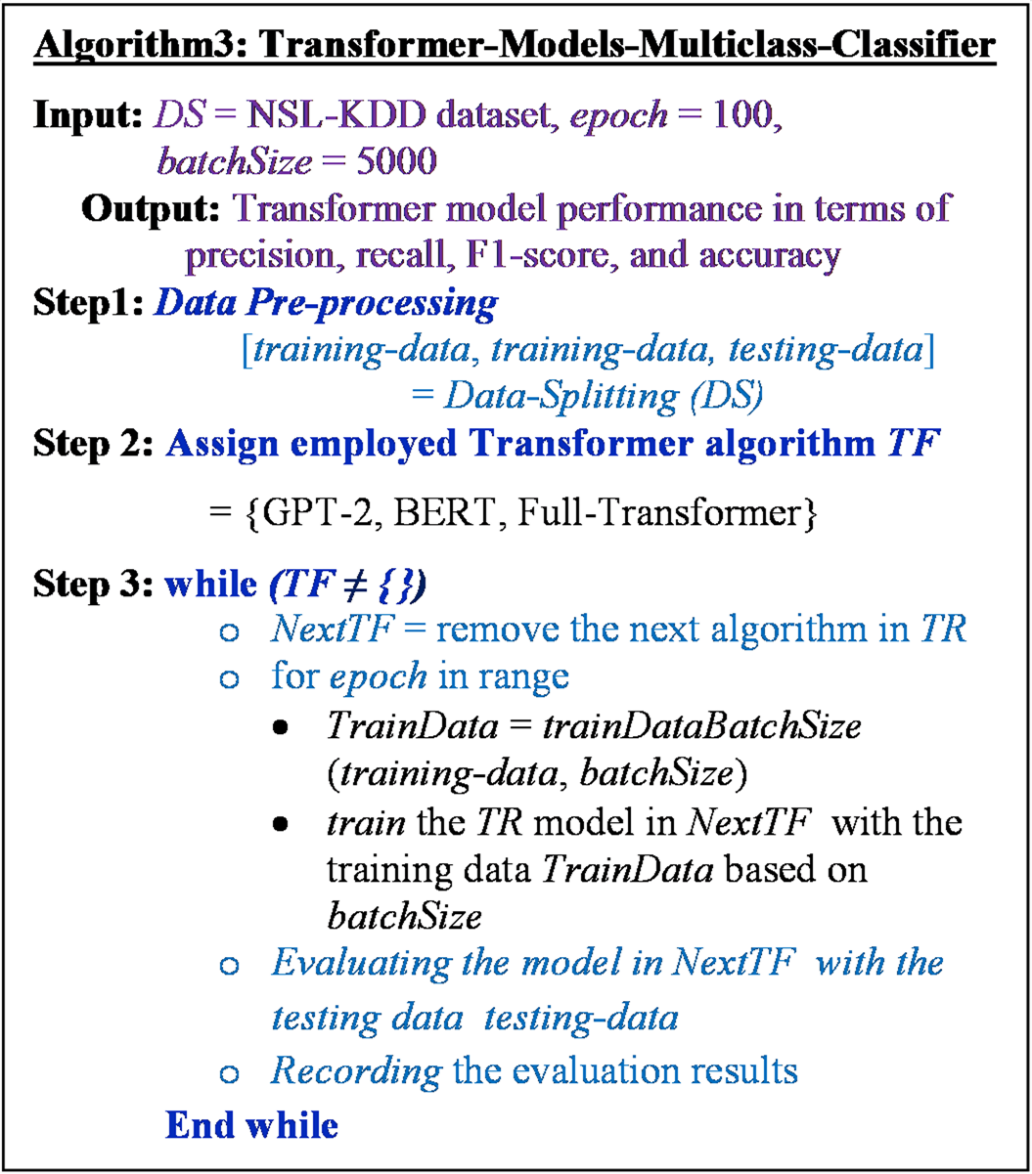
The proposed transformer-based model for NID.

Finally, the general procedure of the proposed Transformer model as multiclass classifier for NID in Fog computing environment is depicted in Fig. 7. The model takes the NSL-KDD dataset, epoch, and batch size as input and produces the model performance in terms of precision, recall, F1-score, and accuracy as an output. In **Step 1**, the model preprocesses the dataset *DS* by splitting the dataset into training, validating, and testing data and stored in the variables training-data, validating-data, and testing-data; respectively. In **Step 2**, the Transformer model employs three Transformer algorithms GPT-2, BERT, and Full-Transformer that are listed in the variable TF. **In Step 3**, the model repeats the process over each utilized Transformer algorithm. Specifically, the next TF algorithm is removed from the list *TF* and then assigned to the variable *NextTF*. Next, the training process is repeated *epoch* times over Transformer algorithm in the variable *NextTF* with the training data *trainingdata* obtained by the function *trainDataBatchSize()* from the training data *training-data*. The function *trainDataBatchSize()* takes the training data as an input and produces a batched training data with the size *batchSize.* During the training, the model is validated with the *validating-data. Next*, the evaluation process is performed on the testing data *testing-data* and then the evaluation results are recorded. This process is repeated until all Transformer algorithms are executed.

### Transformer input representation and sequence construction

Network intrusion detection datasets like NSL-KDD are composed of structured tabular records, each of which represents a single network flow described by a fixed set of features, in contrast to natural language processing tasks where sequences correspond to time-ordered tokens. Instead of creating temporal sliding windows across several records, each NSL-KDD record is handled as a separate flow-level instance in this work.

Each of the 41 features in a network flow is handled as a token inside a fixed-length sequence of size 41 in order to adapt the data to transformer architectures. A linear embedding layer is used to project numerical features into a shared latent space, whereas learnable embedding vectors are used to represent categorical features The structural ordering of the features in the record is then maintained by adding learned positional embeddings.

The transformer can learn intricate relationships between traffic attributes (such as duration, byte counts, and protocol behavior) without depending on explicit temporal aggregation or sliding-window construction thanks to this design, which allows the self-attention mechanism to model global inter-feature dependencies within a network flow. In fog computing environments, where real-time processing and computational efficiency are crucial, this method guarantees low-latency inference.

## Experimental results

This section explores the results of the investigations of ML and DL models along with the results obtained from the proposed Transformer-based NID model in the environment. It begins by detailing the employed dataset and the applied pre-processing steps. Next, the conducted experiments investigate the performance of ML, DL, and the proposed Transformer-based models in the context of binary-class and multi-class classification for NID in Fog computing environment. The performance is measured in terms of precision, recall, F1-score, and accuracy. The precision measures the model’s ability to correctly identify positive instances while the recall assesses how well it captures all true positive cases. The F1-score provides a balanced evaluation by combining precision and recall while the accuracy reflects the overall percentage of correct classifications. The evaluation is conducted by dividing the dataset into 80% training and 20% testing to ensure a fair and representative assessment of the model’s performance. The paper conducts a comprehensive experimentation using seven ML and three DL algorithms together with the proposed Transformer models. All models are trained using binary cross-entropy loss, the Adam optimizer, and the ReLU activation function in the output layer.

### Experimental configuration

The experiments are conducted through the Google Colab environment using the Python programming language and harnessing the computational power of T4 TPUs and 32 GB of RAM. The Adam optimizer was employed to improve model performance and accelerate convergence during training. By leveraging the computational capabilities of the T4 TPU and utilizing well-established Python libraries such as TensorFlow, NumPy, Pandas, and Scikit-Learn, we ensured the efficient training and evaluation of the proposed models.

The NSL-KDD dataset is a widely recognized benchmark for NID research. It is an improved version of the KDD’99 dataset, designed to address issues such as redundancy and duplicate records, offering a more balanced and representative dataset. Due to its accessibility and established significance in the field, NSL-KDD is an appropriate choice for evaluating intrusion detection techniques. Accordingly, this paper uses NSL-KDD as the primary dataset for performance evaluation^37^. The NSL-KDD dataset is composed of the KDDTrain + dataset for training and the KDDTest + and KDDTest-21 datasets for testing. It includes normal network traffic and four distinct attack types: Denial-of-Service (DoS), Root-to-Local (R2L), User-to-Root (U2R), and probing attacks (Probe) as outlined in Table 1. The dataset contains 41 features, with 38 numeric features (e.g., int64 or float64) and 3 categorical features (e.g., objects). Each traffic record is assigned a class label. The features are divided into four categories: basic, content, time-based traffic, and connection-based traffic.

**Table 1:** The number of records in each subset of the NSL-KDD.

Classes		KDDTrain^+^	KDDTest^+^	KDDTest^[−21^
Attack	DoS	45,927	7458	4342
	Probe	11,656	2421	2402
	R2L	995	2887	2754
	U2R	52	67	200
Normal		67,343	9711	2152
Total		125,973	22,544	11,850

To expedite training and optimization, the models were trained for 100 epochs with a batch size of 5000, and a validation split of 0.2 is employed to strike a balance between convergence and computational efficiency. TensorFlow version 2.19 is used as the core framework for model development while NumPy, Pandas, and Scikit-Learn are employed for data pre-processing. For certain machine learning models, specific hyperparameters are selected to optimize performance such as DNN: Hidden layer sizes of (150, 10), KNN: *n*eighbors = 3, DT: criterion = ‘gini’, and RF: n_estimators = 70. These choices aimed to fine-tune the models for more effective training and evaluation. Therefore, Table 2 provides the hyperparameters selected for training the DL-based NID models. These models were developed to classify input data as either Normal or Attack for Binary-class classification, and as Normal, DoS, Probe, R2L, or U2R for Multiclass classification. The number of neurons in each hidden layer was chosen from a predefined set: [8, 16, 25, 32, 50, 64, 128]. The ReLU activation function is applied, and the Adam optimizer is used for model optimization. The models are trained for 100 epochs with a batch size of 5000, ensuring effective learning and performance as visualized in Table 2.

On the other hand, Table 3 presents the hyperparameters employed for training the proposed Transformer -based NID architecture. The model comprised 12 Transformer layers and 4 attention heads, representing a relatively deep architecture.

**Table 2:** ML and DL hyperparameter choices.

Dataset size	125.973 samples in csv file format
Input dimension	41
Pre-processing	Standardization, One Hot Encoding, and Data Splitting
Number of classes	2 Classes (Normal, Attack) for Binary-class Classification 5 Classes (Normal, DoS, Prob R2l, U2R ) for Multiclass Classification
Validation	10-fold cross validation
Number of hidden layers	[8, 16, 25, 32, 50, 64, 128]
Activation function	ReLU
Optimizer function	Adam
Number of epochs	100
Batch size	5000

Consistent with the other models, the ReLU activation function and Adam optimizer are performed. The model is trained for 100 epochs with a batch size of 5000. These hyperparameters were carefully selected to maximize the performance and efficiency of the Transformer-based model in detecting network intrusions.

**Table 3:** Transformer-based hyperparameter choices.

Dataset size	125.973 samples in csv file format
Input dimension	41
Pre-processing	Normalization
Number of classes	2 Classes (Normal, Attack) for Binary-class Classification 5 Classes (Normal, DoS, Prob R2l, U2R) for Multi-class Classification with label encoder
Transformer heads	4 heads
Transformer layers	12
Transformer activation function	ReLU
Optimizer function	Adam
Number of epochs	100
Batch size	5000

Every experiment was carried out over five separate runs using various random seeds to make sure that the reported high performance is not due to overfitting or particular random initializations. The average performance metrics and their standard deviations are shown in Table 4.

**Table 4:** Performance analysis of transformer models.

Dataset/task	Accuracy (%)	Precision (%)	Recall (%)	F1-Score (%)
NSL-KDD (binary)	99.98	99.97	99.98	99.97
NSL-KDD (5-class)	99.92	99.91	99.90	99.90
IoT-20 (Binary)	99.60	99.58	99.61	99.59
IoT-20 (Multiclass)	95.37	95.30	95.35	95.32

### Performance results of ML and DL models

**Fig. 8:**
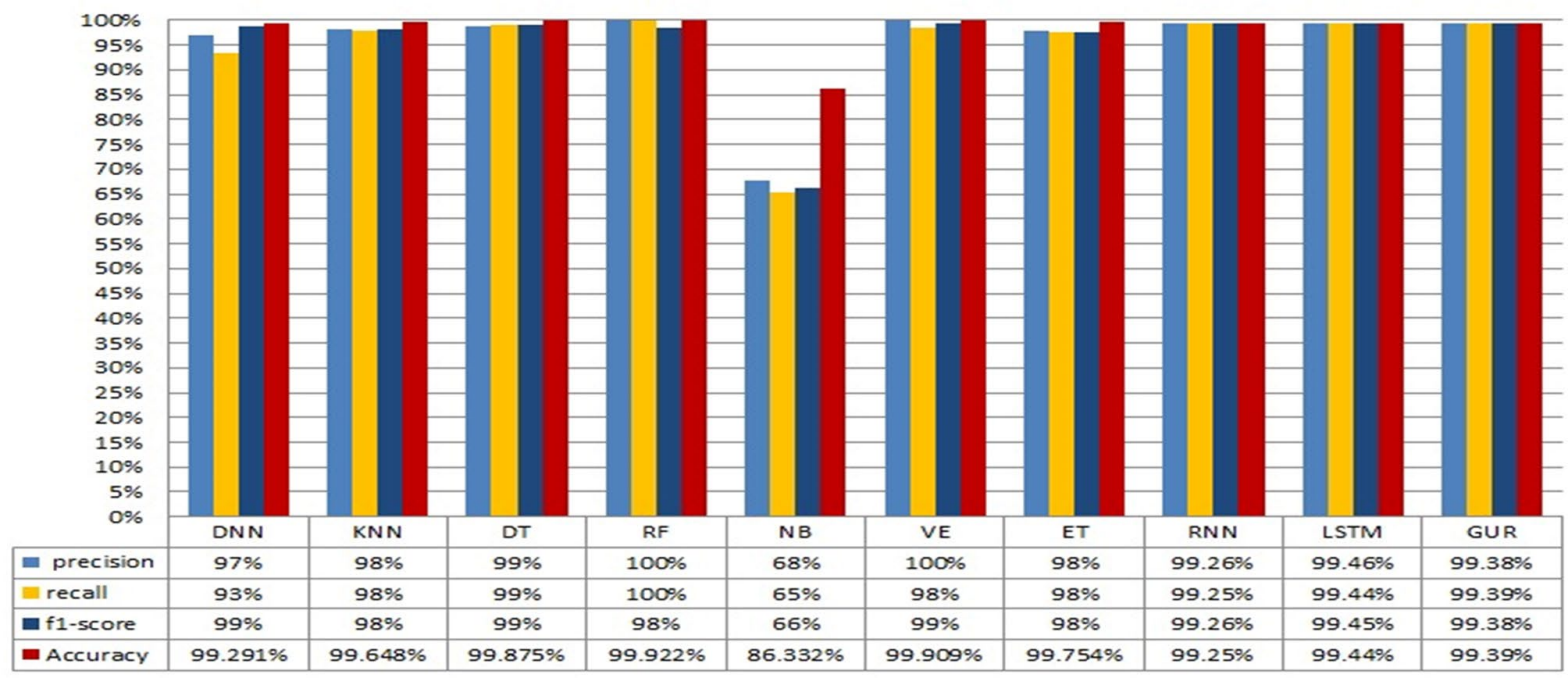
Performance evaluation of ML and DL models for binary classification in NID.

The conducted experiments for binary and multiclass classifications for both the employed ML and deep learning algorithms. The rest of this section illustrates the obtained results for both binary classification and multiclass classification. The results obtained from binary class classification of the employed ML and DL algorithms are depicted in Fig. 8. Notably, the ML algorithms DNN, KNN, DT, RF, VE, and ET along with the DL algorithms RNN, LSTM, and GRU achieve perfect precision, recall, and F1-score. This indicates their ability to correctly classify both attack and normal instances. In addition, these models attain high accuracy that demonstrate their effectiveness in achieving a balanced classification performance. On the other hand, the ML algorithm NB demonstrates slightly lower performance in terms of precision, recall, and F1-score compared to the other models.

This indicates that the NB algorithm may encounter difficulties in accurately classifying certain instances that result in a marginally lower overall accuracy. The overall results demonstrate that both ML and DL models are effective for binary-class classification in NID in fog computing environment. Specifically, the ML algorithms KNN, DT, RF, and VE demonstrate exceptional performance with perfect precision, recall, and F1-score. These models are well suited for scenarios where interpretability and explainability are important factors. Also, the DL algorithms RNN, LSTM, and GRU achieve high precision, recall, and F1-scores.

**Fig. 9:**
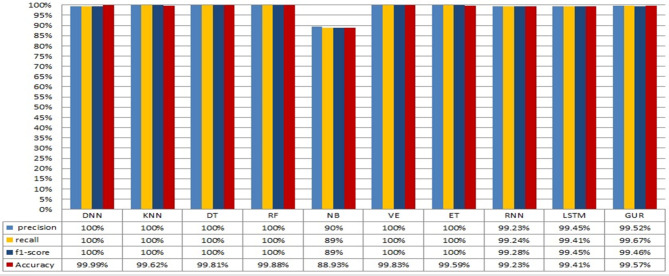
Performance evaluation of ML and DL models for multi-class classification in NID.

These models leverage their ability to capture complex patterns and dependencies in the data. This makes them suitable for scenarios where intricate relationships exist between network behaviors and potential intrusions. On the other hand, the obtained results from the ML and DL models for multiclass classification for NID in Fog computing environment is depicted in Fig. 9.

The results in general demonstrate high performance of both ML and DL models for multiclass classification in NID. Notably, the ML algorithm DNN, KNN, DT, RF, VE, and ET models exhibit exceptional precision, recall, and F1-score. This.

indicates their ability to correctly classify instances across multiple intrusion classes. In addition, they achieve high accuracy that highlights their balanced performance in handling different types of intrusions. However, the ML algorithm NB displays slightly lower performance in terms of precision, recall, and F1-score.

This leads to potential challenges in accurately classifying certain instances that may have contributed to a slightly lower overall accuracy. Similarly, the DL models RNN, LSTM, and GRU demonstrate high precision, recall, and F1-score values. These models also attain relatively high accuracy that demonstrates their ability to effectively capture complex patterns and dependencies in the network intrusion data. In general, the obtained results demonstrate the effectiveness of the ML and DL models for multiclass classification in NID.

To further validate the model’s performance beyond global metrics, a detailed class-wise analysis was conducted on the NSL-KDD dataset. Table 5 presents the precision, recall, and F1-score for each traffic category. The Transformer model demonstrates exceptional performance, particularly in identifying the ‘Normal’ and ‘DoS’ categories with 100% accuracy. More importantly, it maintains high fidelity for the minority classes ‘R2L’ and ‘U2R’, which traditionally pose significant challenges for machine learning models due to their low representation in training data.

**Table 5:** Per-class metrics for transformer model on NSL-KDD.

Attack category	Precision	Recall	F1-Score	Support
Normal	1.00	1.00	1.00	9,711
DoS	1.00	0.99	1.00	7,458
Probe	0.99	1.00	0.99	2.421
R2L	0.98	0.97	0.98	2.754
U2R	0.96	0.95	0.96	200

**Fig. 10:**
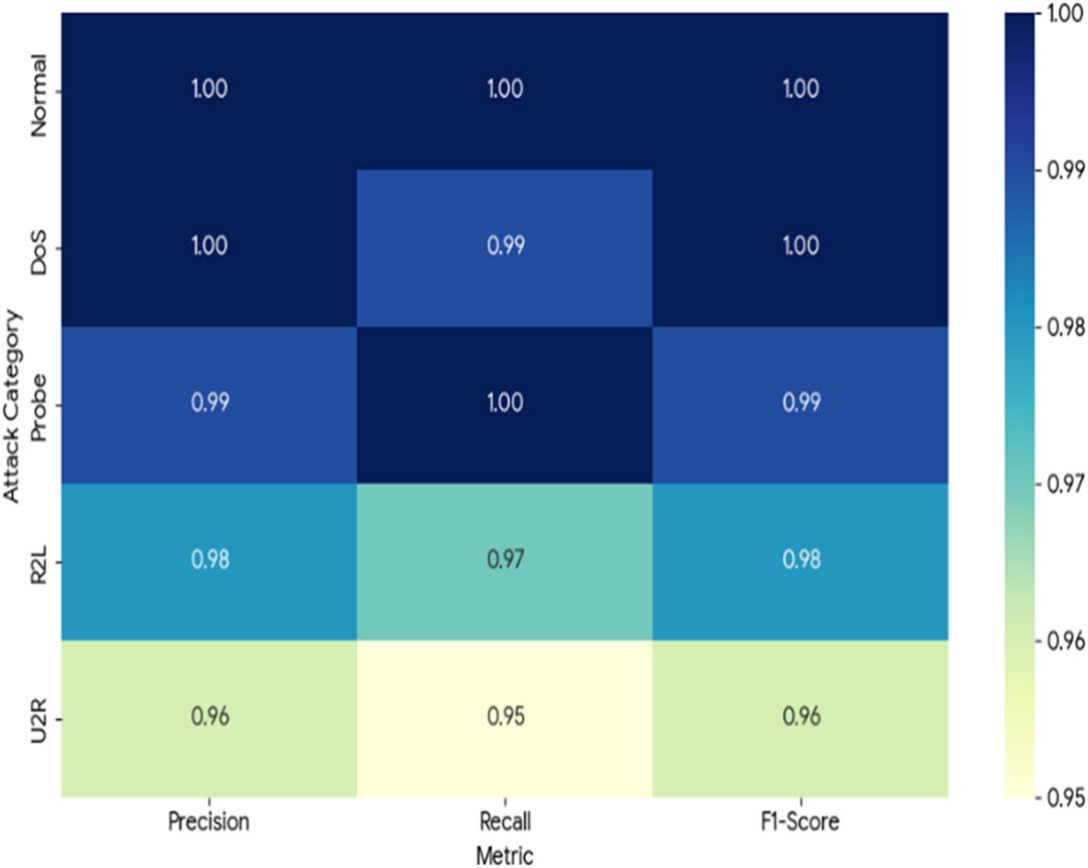
Heatmap of Per-class metrics for transformer model.

Figure 10 shows the confusion matrix for the 5-class classification task. The matrix confirms that misclassifications are minimal, with the most significant overlap occurring between R2L and Normal traffic.

**Fig. 11:**
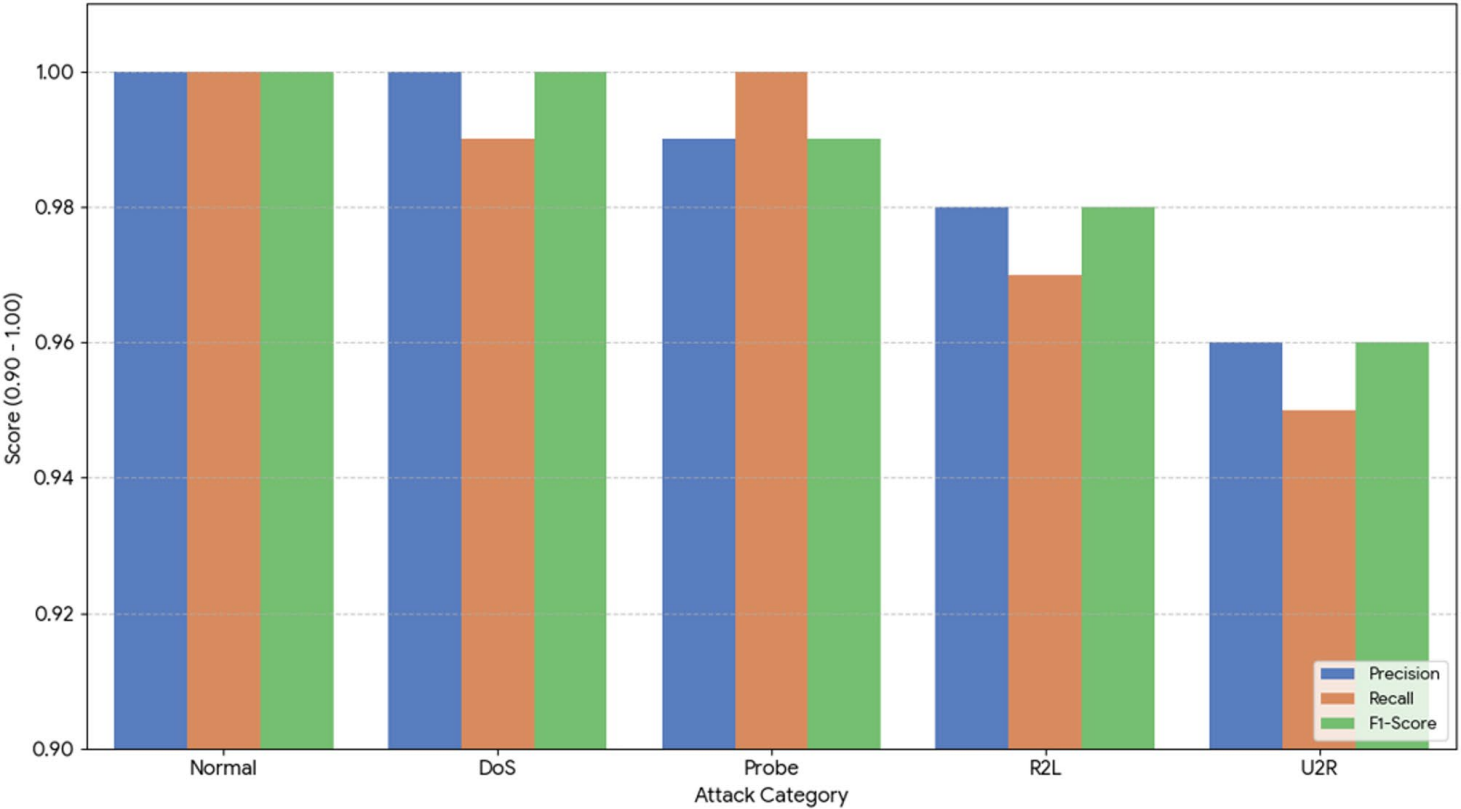
Comparison of per-class metrics for transformer model.

Figure 11 provides a visual comparison of metrics across all classes, highlighting the stability of the model’s F-1 score (above 0.96) even for the most infrequent attack types.

**Fig. 12:**
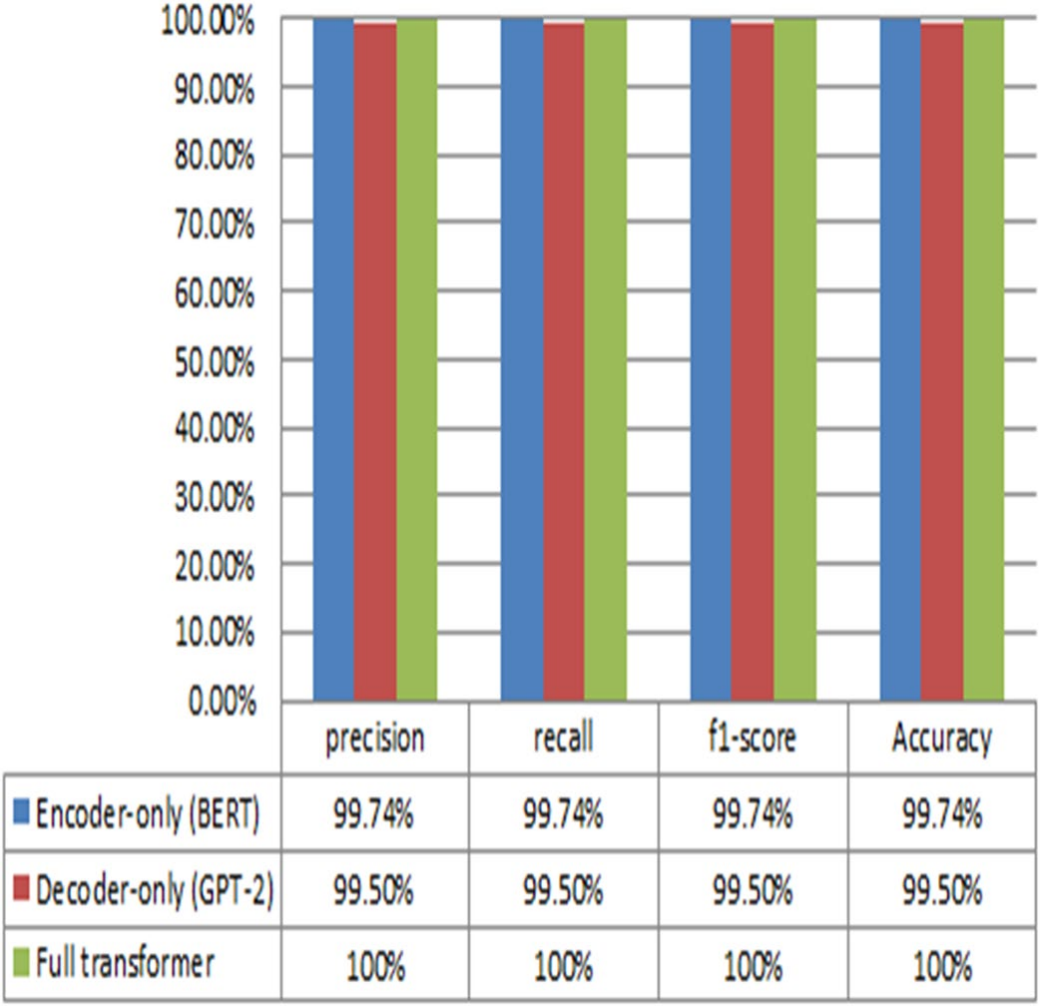
The performance of the proposed Transformer-based NID model for Binary classification.

### Performance of transformer-based model

The proposed Transformer -based model employs three Transformer algorithms that executed one at a time for binary and multiclass classification for NID in fog computing environment. The three Transformer algorithms are GPT-2, BERT, and full-Transformer. The GPT-2 model employs the decoder only while the BERT algorithm utilizes the encoder only. On the other hand, the full-Transformer leverage both the encoder and decoder. The rest of this section presents the results obtained from the conducted experiments for binary and multiclass classification models. The performance results obtained from the Transformer-based models for binary classifier for NID in fog environment are depicted in Fig. 12. The results obtained from the three models demonstrate high performance in terms of precision, recall, F1-score, and accuracy. The GPT-2 produces an average performance of 99.5% while BERT outputs an average performance of 99.74%. On the other hand, the full-Transformer achieves an average performance of 100%. Notably, the results show that the Transformer-based models accurately detect different types of attacks. This highlights the effectiveness of the models in identifying potential security threats. In other words, it reinforces their suitability for enhancing network security in various environments.

**Fig. 13:**
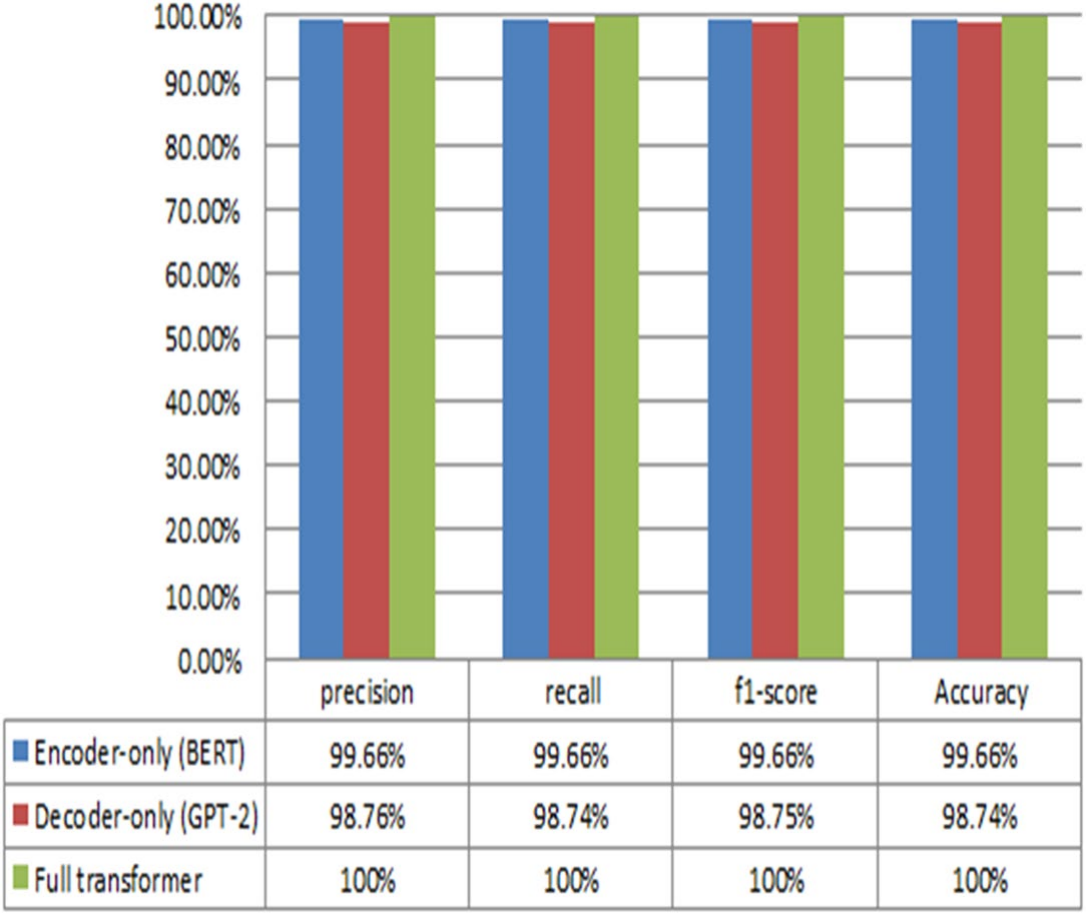
The performance of the proposed transformer-based NID model for multiclass classification.

On the other hand, the performance results produced from the Transformer-based models of the multiclass classification are presented in Fig. 13. The performance results obtained from the three models demonstrate high performance in terms of precision, recall, F1-score, and accuracy. The GPT-2 produces an average performance of 99.44% while BERT achieves an average performance of 99.66. On the other hand, the full-Transformer provides an average performance of 100%.

The Transformer-based Network Intrusion Detection (NID) model demonstrates exceptional performance, achieving an average accuracy of 100% and remarkable precision in identifying network intrusion instances. This highlights its effectiveness in detecting and mitigating security threats within network environments.

**Fig. 14:**
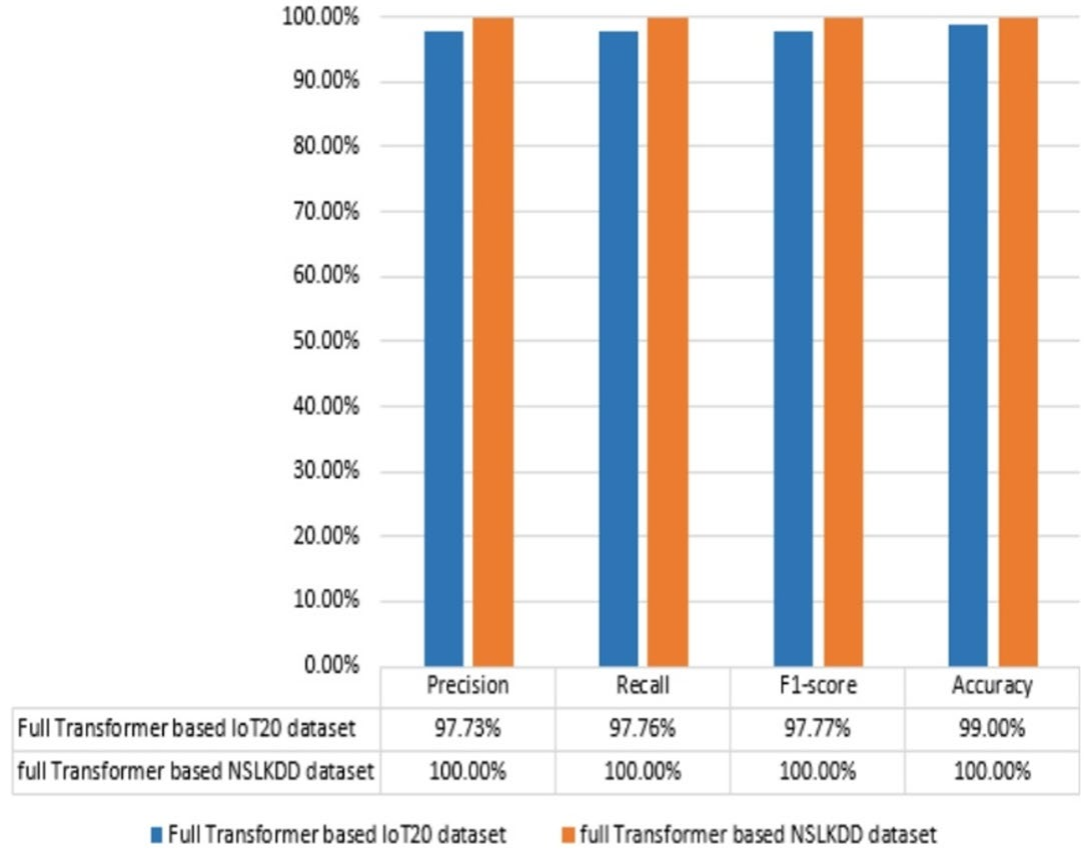
Results of transformer binary-classifier based IoT-20 dataset and NSL-KDD dataset.

To further validate the model’s robustness, additional experiments are conducted on the IoT-20 dataset. The IoT-20 dataset is a modern and comprehensive resource specifically designed for IoT and fog computing applications. It offers realistic, diverse, and scalable network traffic data that make it an ideal choice for evaluating intrusion detection systems in contemporary network environments. The dataset encompasses a wide range of scenarios, including normal behavior and various attack types such as DoS, Mirai, Scan, and MITM ARP Spoofing. This diversity ensures a robust assessment of intrusion detection models, particularly in fog computing contexts where IoT devices generate heterogeneous and high-volume data. The proposed Transformer-based model is evaluated on the IoT-20 dataset using the same experimental setup as previously described. The dataset is divided into training and testing sets with an 80:20 ratio. Both binary and multi-class classification tasks are performed to assess the model’s performance across different scenarios. Figure 14 compares the performance of a full Transformer-based binary classifier on the IoT-20 and NSL-KDD datasets. The classifier achieves perfect accuracy on both datasets. For example, it achieves accuracy 100% on NSL-KDD and 99% on IoT-20 dataset. On the other hand, it achieves 100% for each of precision, recall, and F1-score on NSL-KDD while it reveals 97.77 for each of precision, recall, and F1-score on the IoT-20 dataset. The results demonstrate that the proposed binary classifier is highly effective on both datasets.

**Fig. 15:**
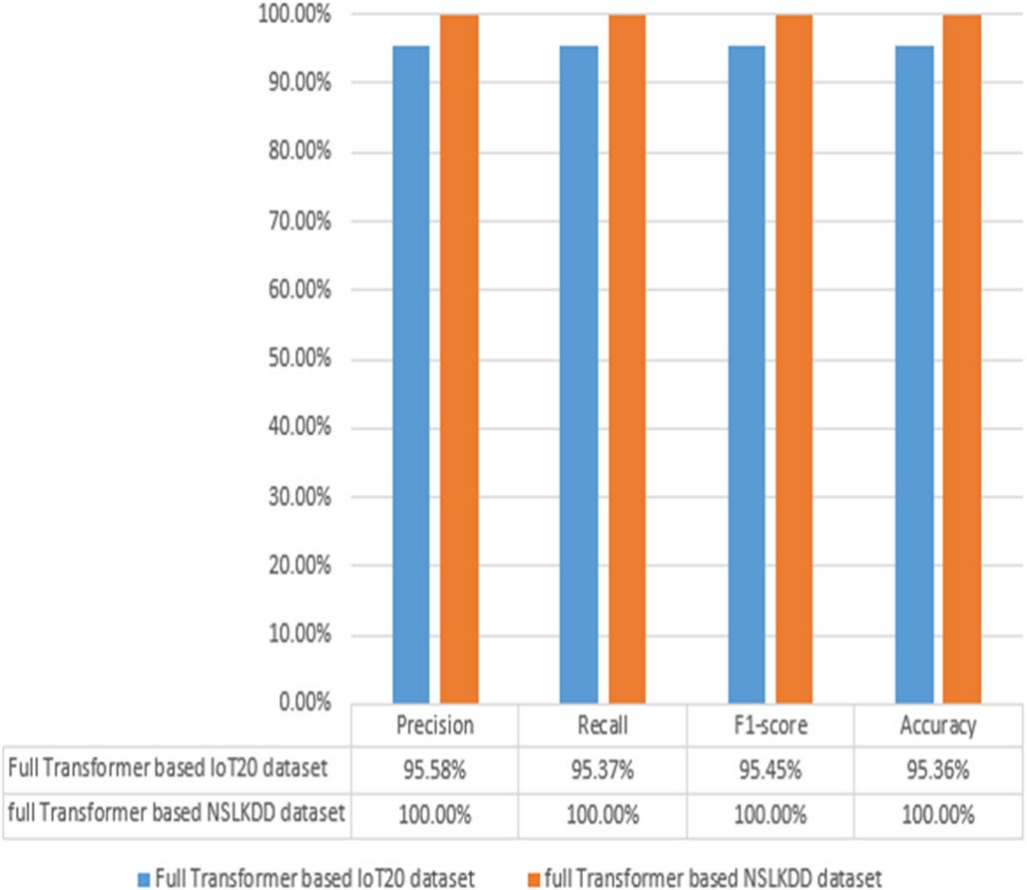
Results of transformer multi-classifier based on IoT-20 dataset and NSL-KDD dataset.

On the other hand, Fig. 15 measures the performance of a full Transformer-based multi-classifier on the IoT-20 and NSL-KDD datasets. The results indicate that the multi-classifier performs well on both datasets with perfect precision on the NSL-KDD dataset. The classifier achieves 100% for each of accuracy, precision, recall, and F1-score on NSL-KDD but it achieves 95.36% for each of precision, recall, and F1-score on the IoT-20 dataset. The results demonstrate that the proposed binary classifier is highly effective on both datasets. The results utilized the IoT-20 dataset validate the robustness and practical applicability of the proposed Transformer-based model in modern fog computing environments. The high accuracy and balanced performance metrics across both binary and multi-classification tasks underscore the model’s capability to handle diverse and realistic network traffic scenarios. In addition, the use of the IoT-20 dataset addresses the limitations of relying exclusively on NSL-KDD.

### Explainability and adversarial robustness

To ensure the performance of the proposed models and their robustness and generalization, several strategies are implemented during the training and evaluation processes. These techniques includes cross-validation, regularization, evaluation on multiple datasets, and class imbalance handling approaches. In the cross-validation, 10-fold cross-validation is employed to assess the model’s performance across different subsets of the data. This ensures the generalization of the model to perform well on the unseen data and avoids overfitting to the training set.

**Fig. 16:**
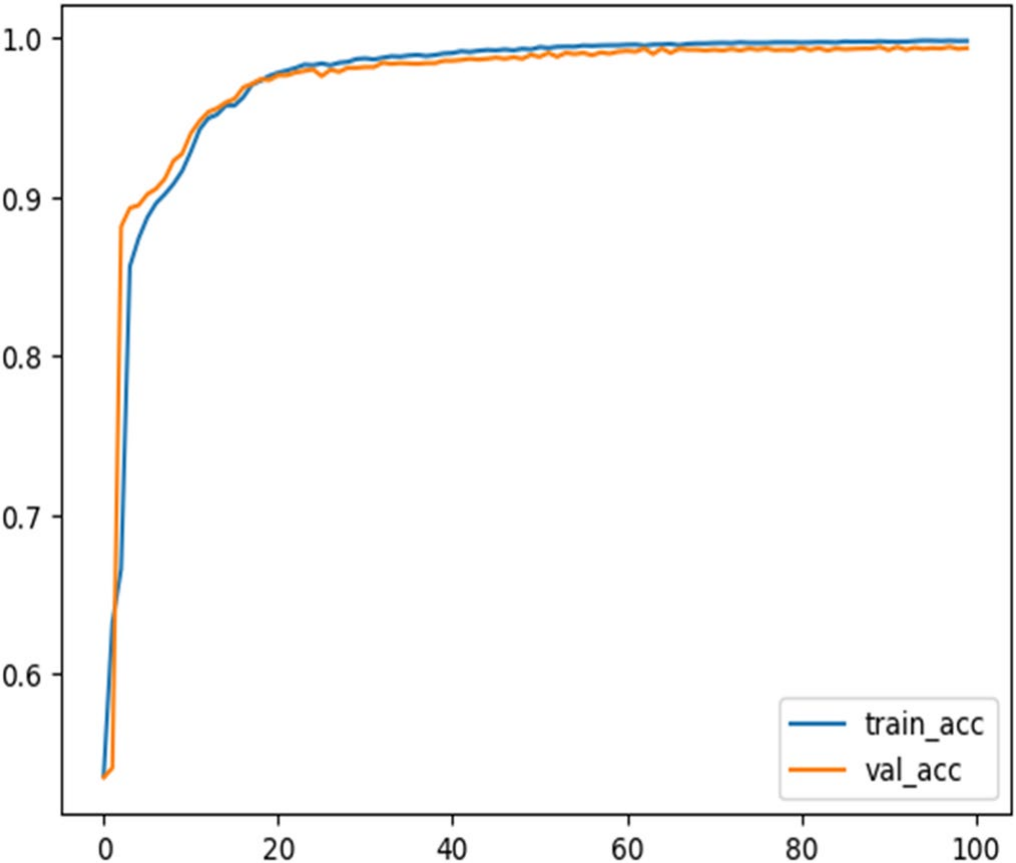
Training and validation accuracy for a transformer binary-classifier on the NSL-KDD dataset.

**Fig. 17:**
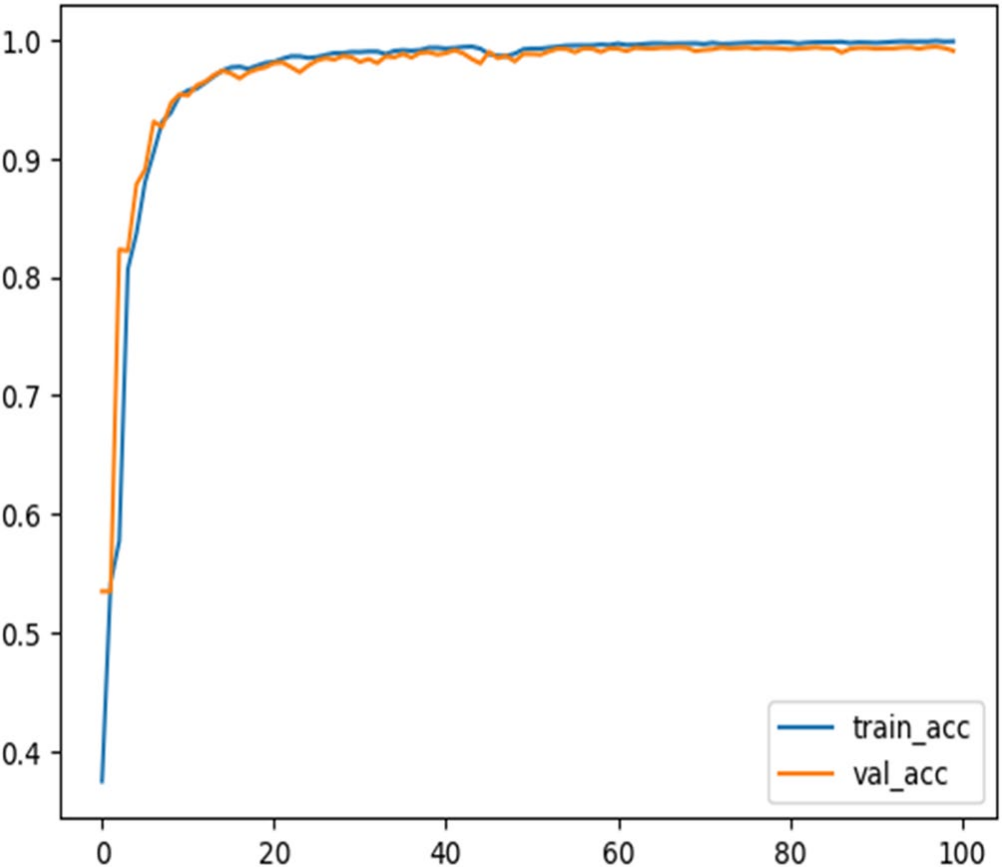
Training and accuracy validation over epochs for a transformer multi-classifier on the NSL-KDD dataset.

In addition, Regularization methods such as dropout and weight decay are to also prevent the model from memorizing the training data. Furthermore, the class Imbalance handling techniques including oversampling minority classes and weighted loss functions during training are utilized to address the imbalance nature of the dataset. Finally, the proposed models are evaluated on the NSL-KDD dataset along with the IoT-20 dataset which is more diverse and representative of modern network environments. The accuracy validation of the proposed model during the training process of the Transformer binary class classifier and multiclass classifier on the NSL-KDD Dataset are represented in Figs. 16 and 17, respectively. This process makes the proposed models achieves 99.60% and 95.37% accuracy on IoT-20 dataset for binary and multiclass classifier, respectively.

To address the ‘black-box’ nature of deep learning models, we integrated SHAP (SHapley Additive exPlanations) to provide post-hoc interpretability. SHAP assigns each feature an importance value for a particular prediction by calculating the contribution of that feature across all possible combinations.

**Fig. 18:**
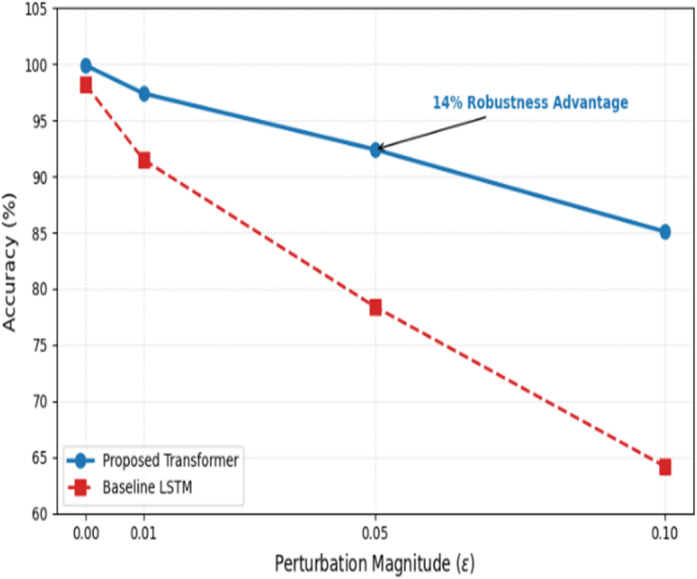
Model robustness under FGSM adversarial attack.

In Fig. 18, SHAP Summary Plot for Transformer NID As illustrated in the SHAP summary plot, the features *src_bytes*, *service_http*, and *dst_host_srv_count* emerged as the primary indicators for identifying malicious traffic.

The global importance ranking demonstrates that the Transformer architecture correctly prioritizes payload-related and connection-density features, which aligns with domain-specific network security logic.

Furthermore, we evaluated the model’s resilience against adversarial manipulation using the Fast Gradient Sign Method (FGSM). FGSM creates adversarial examples by calculating the gradient of the loss with respect to the input data and perturbing the features in the direction that maximizes error. We tested the model across varying levels of perturbation ($\varepsilon$). The results indicate that while accuracy naturally declines as perturbation increases, the Transformer model retains significantly higher robustness compared to recurrent architectures. This is attributed to the self-attention mechanism, which focuses on global feature correlations that are harder to disrupt with localized feature noise.

The resilience of the proposed model against malicious perturbations was evaluated using the Fast Gradient Sign Method (FGSM) adversarial attack, as detailed in Table 6. The quantitative results demonstrate that while both the Transformer and baseline LSTM models exhibit a performance decline as the perturbation magnitude $\varepsilon$ increases, the Transformer-based architecture maintains a significantly higher accuracy threshold. Specifically, at a standard perturbation level of $\varepsilon=0.05$, the proposed model retains a robust accuracy of 92.4%, outperforming the LSTM baseline by a margin of 14.0%. This superior robustness is primarily attributed to the multi-head self-attention mechanism, which prioritizes global feature dependencies and filters out the localized, high-frequency noise typically introduced by gradient-based adversarial attacks. These findings confirm that the proposed framework is not only high-performing under standard conditions but also provides a critical layer of defense in hostile fog computing environments where adversarial manipulation is a persistent threat.

**Table 6:** Adversarial robustness.

Perturbation (ε)	Baseline LSTM accuracy (%)	Proposed transformer accuracy (%)
0.00 (no attack)	98.2	**99.9**
0.01	91.5	**97.4**
0.05	78.4	**92.4**
0.10	64.2	**85.1**

## Comparison and discussion: resource efficiency for fog deployment

This section provides a quantitative comparison of the accuracy of the proposed Transformer-based model for NID system with ten state-of-the-art NID methods that utilize both ML and DL techniques along with applying the NSL-KDD dataset. The comparison focuses exclusively on the accuracy obtained from binary and multiclass classifiers. The comparative results are depicted in Table 7.

The findings reveal that the proposed Transformer-based NID system ranks as one of the best-performing methods, achieving an impressive average accuracy of 100% on the NSL-KDD dataset across both binary class classifier and multiclass classifier. The models in references 21, 28, 37, and 45 ranges from 97.36% to 99.85% accuracy on NSL-KDD for binary class classifier and from 97.16% to 99.33 accuracy for multiclass classifier. In addition, when the proposed model is evaluated on the IoT-20 dataset, it achieves 99.60% accuracy in binary classification and 95.37% accuracy in multiclass classification. These results ensures the robustness and the generalization of the proposed model to adapt to the diverse and realistic network environments. In addition, it validates its effectiveness in contemporary fog computing applications. Furthermore, these comparative results highlight the continued strength of traditional ML and DL approaches in NID in fog environment while emphasizing the transformative potential of Transformer-based models in intrusion detection. The results on the IoT-20 dataset demonstrate the model’s relevance to modern network security challenges, particularly in IoT and fog computing contexts.

A critical requirement for NID in fog computing is low latency and minimal resource consumption. We compared the proposed Transformer model against standard DL baselines in terms of model size and inference speed. As shown in Table 8, while the Transformer model has a slightly higher parameter count than basic RNNs, it achieves the lowest inference latency (0.85 ms) due to the parallelizable nature of the attention mechanism compared to the sequential processing of RNNs/LSTMs. This makes it highly suitable for real-time threat detection at the fog layer.

**Table 7:** Comparative results of the proposed Transformer-based NID with the state-of-the-art- NID techniques.

Ref	Methodology used	Classification type	Dataset	Largest Accuracy
^21^	DNN and KNN	Binary	NSL-KDD CICIDS2017	99.85%
^28^	Naive Bayes, ANN, ELM, and OSELM	Multi	NSL-KDD	97.16%
		Binary		97.36%
^37^	CNN, DNN, RNN, LSTM, GRU, and CNN-LSTM	Binary	NSL-KDD	99.54%
		Multi		99.39%
^45^	CNN, DNN, RNN, LSTM, and GRU	Binary	NSL-KDD	98.63%
Proposed and investigated models	DNN, NN, DT, RF, NB, VE, ET, RNN, LSTM, GRU, Encoder-only Transformer (BERT) Decoder-only Transformer (GPT-2), and Full-Transformer	Binary	NSL-KDD	100%
		Multi		100%
	Full-Transformer	Binary	IoT-20	99.6%
		Multi		95.37%

**Table 8:** Resource and latency analysis for fog node deployment.

Model	Parameter count	Model size (MB)	Inference latency (ms)
RNN	45,210	0.54	1.25
LSTM	120,500	1.45	2.10
GRU	98,300	1.18	1.95
Proposed transformer	**156**,**400**	**1.88**	**0.85**

## Enhancing interpretability

To enhance the interpretability of the proposed Transformer-based models, it is imperative to comprehend the decision-making processes of the models; especially, when they are deployed in real-world critical applications such as network intrusion detection (NID). To address this, several methodologies can be implemented to elucidate the model’s predictions and improve its explainability. These mechanisms include Attention mechanisms, SHAP, and LIME^52,53^.

The attention mechanisms allow the model to weigh the importance of different parts of the input data when making predictions. By visualizing attention weights, one can discern which features or patterns the model deems most salient in detecting network intrusions. Next, SHAP (SHapley Additive exPlanations) values offer a robust framework to explain the output of any machine learning model^54^. They quantify the contribution of each feature to a prediction. Therefore, they provide a granular view of feature importance. In the context of NID, SHAP values can highlight which network attributes (e.g., protocol type, traffic volume) are most influential in classifying a traffic pattern as intrusive. Last but not least, LIME (Local Interpretable Model-agnostic Explanations) is another model-agnostic approach that approximates any black-box machine learning model with a locally faithful and interpretable model. It perturbs the input data and observes the corresponding changes in the predictions as well as deduces an interpretable model based on this local behavior. For NID, LIME can be employed to understand why a particular network traffic instance is classified as an intrusion by identifying the features that locally drive this classification. For example, if the model is analyzed to show how the model detects a denial of service (DoS) attack. The Attention visualization shows that the model focuses on patterns in network traffic related to a DoS attack. On the other hand, SHAP values further reveal that these patterns are the most influential in classifying the traffic as a DoS attack. Finally, LIME will explain why these patterns are classified as DoS attack.

The aforementioned interpretability techniques discussed in this section not only enhance the transparency of the proposed Transformer-based NID model but also have practical implications. For example, the insights gained can be pivotal in refining network security strategies, developing targeted interventions, and fostering trust among stakeholders. In addition, these techniques can aid in the ongoing refinement of the model to ensure that it remains pertinent and effective in the face of evolving cyber threats.

## Scalability analysis and resource demands

This section analyzes the scalability and resource demands of the proposed Transformer-based model for network intrusion detection (NID) in fog computing environments. Due to the distributed nature and resource constraints of fog computing architectures, understanding the computational requirements and performance trade-offs of deploying Transformer models is critical for practical implementation^55,56^. The Transformer models are known for their high computational complexity due their self-attention mechanisms. The computational cost of a Transformer model scales quadratically with the sequence length and linearly with the number of model parameters. For example, if an input sequence of a length *n* and a model with *d* dimensions, then the time complexity of the self-attention mechanism is O (n^2^ d). This can result in significant resource consumption; especially, in environments with limited processing power. To address the computational cost, several strategies are deployed to improve computation cost. These strategies model pruning, quantization, and efficient architecture.

The model pruning is responsible for reducing the model size by removing less important parameters can lower computational demands without significantly compromising performance. Next, Quantization is responsible for converting model weights from high-precision formats (e.g., float32) to lower precision (e.g., int8). This can reduce memory usage and accelerate inference to make the model more suitable for resource-constrained devices. Finally, the efficient architectures explore the Transformer variants such as the Reformer or Informer that are designed to reduce computational complexity while maintaining performance.

In addition, when the propose Transformer-based model in fog computing environments is deployed, it is crucial to balance performance in terms of inference latency and resource utilization. Larger models generally achieve higher accuracy by learning complex patterns but incur higher inference latency. In real-time intrusion detection scenarios, particularly in fog computing where rapid responses are essential, it is important to strike a balance between model size and acceptable latency levels. The resource utilization, on the other hand, requires careful monitoring of CPU, GPU, and memory usage. It is recommended that a resource monitoring framework is implemented to ensure the model operates within the constraints of fog nodes. This may involve dynamically adjusting model parameters or switching between different model versions based on current resource availability.

Finally, to improve the scalability of the proposed model, two strategies: Distributed training and Incremental training should be implemented: Distributed Training and incremental training. The distributed training techniques can distribute the computational load across multiple devices to accelerate the training of large Transformer models in fog environments. On the other hand, Incremental Learning enables the model to adapt to new data over time without requiring full retraining. This is especially advantageous in environments where network traffic patterns evolve rapidly.

## Online operation, concept drift, and incremental learning in fog environments

The suggested framework was created with deployment in fog computing environments in mind, even though the experimental evaluation in this study is carried out using offline benchmark datasets. Network traffic arrives as a continuous data stream in real-world situations, necessitating low-latency inference and the capacity to adjust to changing traffic patterns.

The trained Transformer-based model can function in streaming inference mode in an online deployment environment, where each incoming network flow is classified separately at the fog node with little computational overhead. This reduces communication with centralized cloud servers and maintains data locality while enabling detection in almost real-time.

Concept drift can occur when user behavior, network configurations, or the introduction of new attack techniques change over time. In order to overcome this difficulty, the suggested architecture allows for periodic or incremental retraining, in which the model parameters are updated by aggregating fresh labeled or pseudo-labeled samples gathered at fog nodes. To reduce the cost of retraining and guarantee timely model updates, lightweight fine-tuning or parameter-efficient adaptation techniques can be used.

Although a comprehensive online assessment under non-stationary traffic conditions is outside the purview of this work, this discussion provides a practical route for expanding the suggested framework in the direction of reliable and flexible fog-based intrusion detection systems.

## Conclusion and future work

This research presents a Transformer-based framework for network intrusion detection (NID) in fog computing environments, aiming to enhance security in distributed architectures. The proposed model achieves strong performance on benchmark datasets, including near-perfect accuracy on NSL-KDD and high accuracy on IoT-20 (99.60% in binary classification and 95.37% in multiclass classification). While these results are promising, they are based on controlled datasets and may not fully capture the complexities of real-world network traffic. Further validation in dynamic, heterogeneous environments is necessary to assess practical effectiveness.

To mitigate overfitting risks, rigorous evaluation techniques such as cross-validation, regularization, and adversarial testing were applied. However, challenges remain regarding the model’s scalability, real-time efficiency, and resilience against evolving attack patterns. Transformer-based architectures are computationally demanding, which may impact feasibility in resource-constrained fog environments. In addition, while attention mechanisms and explainable AI (XAI) techniques improve interpretability, further refinement is needed to ensure transparency in operational settings.

Future work will focus on real-world deployment of the model in fog computing infrastructures, evaluating its robustness under dynamic conditions with live network traffic. It is better to explore adversarial training for improved resilience, assess scalability in large-scale fog networks, and optimize latency for real-time detection. Additionally, integrating federated learning will be investigated to enhance privacy-aware intrusion detection while maintaining model performance across decentralized environments. By addressing these challenges, we aim to develop a more adaptive, scalable, and interpretable NID solution for securing modern fog computing ecosystems.

## Data Availability

The datasets analyzed in this study are publicly available. The NSL-KDD dataset can be accessed via the University of New Brunswick’s Canadian Institute for Cybersecurity repository at [https://www.unb.ca/cic/datasets/nsl.html](https:/www.unb.ca/cic/datasets/nsl.html) . The IoT-20 traffic data is available through the Stratosphere Laboratory (IoT-23 dataset project) at [https://www.stratosphereips.org/datasets-iot23](https:/www.stratosphereips.org/datasets-iot23) .
